# Fosl2 Regulates FSH‐Dependent Follicle Maturation Through Feedback Amplification of FSH/FSHR Signaling

**DOI:** 10.1002/advs.75223

**Published:** 2026-04-09

**Authors:** Hongru Shi, Chaoli Chen, Zaohong Ran, Jianning Liao, Zian Wu, Xiaodong Wang, Yongheng Zhao, Wenkai Ke, Bowen Tan, Yun Liu, Youqiang Su, Wei Ren, Xiang Li, Changjiu He

**Affiliations:** ^1^ Key Laboratory of Agricultural Animal Genetics, Breeding and Reproduction of Ministry of Education College of Animal Sciences and Technology Shennongjia Science and Technology Innovation Center, Shennongjia Field Comprehensive Scientific Observation and Research Station of the Ministry of Agriculture and Rural Affairs Huazhong Agricultural University Wuhan P. R. China; ^2^ Department of Obstetrics Maternal and Child Health Hospital of Hubei Province Wuhan P. R. China; ^3^ Shandong Provincial Key Laboratory of Animal Cell and Developmental Biology School of Life Sciences Shandong University Qingdao P. R. China

**Keywords:** estradiol, follicle, *Fosl2*, FSH, granulosa cell

## Abstract

Follicle stimulating hormone (FSH)‐dependent follicle maturation constitutes the cornerstone of female reproductive cyclicity and fertility, with FSH/FSHR signaling recognized as the regulator. While amplification of this signaling is essential for FSH‐dependent follicle maturation, the molecular drivers remain less well‐understood. Through integrated single‐cell and spatial transcriptomic analyses, we identified *Fosl2* as an FSH‐responsive transcription factor exhibiting a dynamic temporal expression pattern that closely mirrored that of the *Fshr*. In vitro *Fosl2* knockdown resulted in notable reductions in granulosa cell proliferation, induced apoptosis, and disrupted FSH‐dependent follicle maturation. In vivo studies using conditional *Fosl2* knockout demonstrated a complete halt in FSH‐dependent follicle maturation and resultant infertility. Mechanistic exploration unveiled that FSH/FSHR initiates *Fosl2* transcription via the cAMP‐PKA‐CREB cascade, while FOSL2 protein, in turn, acts as a direct transcriptional activator of the *Fshr* gene itself, as well as estrogen‐synthesis genes (*Cyp11a1* and *Cyp19a1*), thereby establishing a positive feedback loop for FSH/FSHR signaling. Cross‐species validation demonstrated evolutionary conservation of this loop, with *Fosl2* knockdown impairing FSH/FSHR signaling in sheep and human. Our findings identify a *Fosl2*‐centered feedback loop essential for amplifying FSH/FSHR, underscoring *Fosl2*’s critical role in reproduction.

## Graphical abstract

This study identifies a FOSL2‐driven positive feedback loop that amplifies FSH/FSHR signaling. During FSH‐dependent follicle maturation, FSH induces *Fosl2* expression via the cAMP‐PKA‐CREB cascade. FOSL2 in turn binds the promoters of *Fshr* and estrogen‐biosynthesis genes to enhance their transcription, thereby increasing *Fshr* mRNA level and amplifying FSH/FSHR signaling.

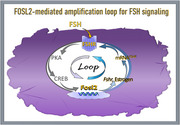

## 1 Introduction

Subfertility and infertility pose major global health burdens, with defective folliculogenesis recognized as a critical etiological factor [1, 2]. Folliculogenesis initiates with primordial follicle activation and progresses sequentially through primary, secondary, small antral, and large antral stages prior to ovulation competency acquisition. A critical developmental milestone occurs with the emergence of the follicular antrum: pre‐antral folliculogenesis is governed by FSH‐independent mechanisms, whereas subsequent FSH‐dependent stages require precise orchestration by FSH and luteinizing hormone (LH) [3, 4, 5]. The FSH‐dependent follicle maturation, characterized by rapid follicular antrum expansion, underpins fundamental reproductive processes through estrogen release. These processes include the initiation of reproductive cycles, maintenance of secondary sexual characteristics, generation of libido, and endometrial growth. Clinically, its dysregulation is a prevalent cause of female infertility and is pathologically linked to disorders such as menstrual irregularities, polycystic ovary syndrome (PCOS), luteinized unruptured follicle syndrome, and amenorrhea [6, 7].

During FSH‐dependent follicle maturation, FSH binds its receptor (FSHR) on mural granulosa cells (GCs). This binding predominately activates the cAMP‐PKA cascade, driving GC proliferation, apoptosis suppression, estrogen biosynthesis, and antrum expansion [8]. At the molecular level, PKA phosphorylates CREB and histone H3 to transactivate CRE‐containing genes. For non‐CRE targets, FSH engages alternative pathways including PI3K‐AKT [9, 10, 11]. FSH potentiates AKT phosphorylation indirectly, eliciting pleiotropic effects: (i) mTOR activation to modulate transcription and translation [12, 13]; (ii) promotion of GC proliferation and estrogen synthesis via GSK3*β* inhibition, relieving suppression of CyclinD2 and *β*‐catenin [14]; and (iii) FOXO1/3 inactivation to attenuate their anti‐proliferative and pro‐apoptotic functions [15, 16]. Emerging evidence further implicates FSH in modulating ERK, Wnt, p38MAPK, and Sgk pathways [14, 17, 18, 19, 20], collectively ensuring follicle survival and maturation.

Throughout the FSH‐dependent phase (a period lasting approximately 48 h in mice), FSH/FSHR signaling exhibits dynamic modulation rather than sustained activity [21, 22, 23, 24]. Initial low‐intensity signaling (0–12 h) supports preparatory processes—nucleobase synthesis, DNA replication, and mRNA processing—without overt morphological changes. A pivotal transition occurs at 12–24 h, marked by pronounced FSH/FSHR signaling amplification. This amplification triggers rapid follicular maturation characterized by significant antrum expansion, accelerated GC proliferation, and peak estrogen synthesis, culminating in ovulation competence acquisition [22]. FSH/FSHR amplification is indispensable for FSH‐dependent follicle maturation. Although its molecular drivers remain incompletely defined, transcriptional upregulation of *Fshr* constitutes a critical requirement [24]. Current evidence suggests the presence of GC‐intrinsic self‐amplifying loops, wherein initial FSH signaling induces specific transcription factors (TFs; e.g., SF‐1, estrogen receptors), which subsequently enhance *Fshr* expression, thereby amplifying FSH/FSHR [24, 25, 26]. These loops govern the small antral‐to‐preovulatory follicle transition. Thus, identifying FSH‐responsive TFs driving them is fundamental to elucidating the molecular basis of FSH/FSHR amplification.

Through integrated single‐cell and spatial transcriptomic analyses, this study identifies FOS‐like antigen 2 (*Fosl2*), also known as FRA‐2, as an FSH‐inducible TF that exhibits a dynamic temporal expression pattern that closely mirrored that of the *Fshr*. As a member of the AP‐1 TF family, *Fosl2* has been implicated in diverse biological processes, including bone and heart development, immune regulation, and circadian rhythms [27, 28]. Here, we demonstrated that conditional knockout of *Fosl2* in GCs disrupts FSH‐dependent follicle maturation, causing reproductive cyclicity loss and infertility. Mechanistically, *Fosl2* deficiency impaired *Fshr* transcriptional upregulation and abolished estrogen synthesis, thereby disrupting the self‐amplifying loop of FSH/FSHR.

## 2 Results

### 2.1 *Fosl2* Identified as an FSH‐Responsive TF With a Dynamic Temporal Expression Pattern That Closely Mirrored That of the *Fshr*

**FIGURE 1:**
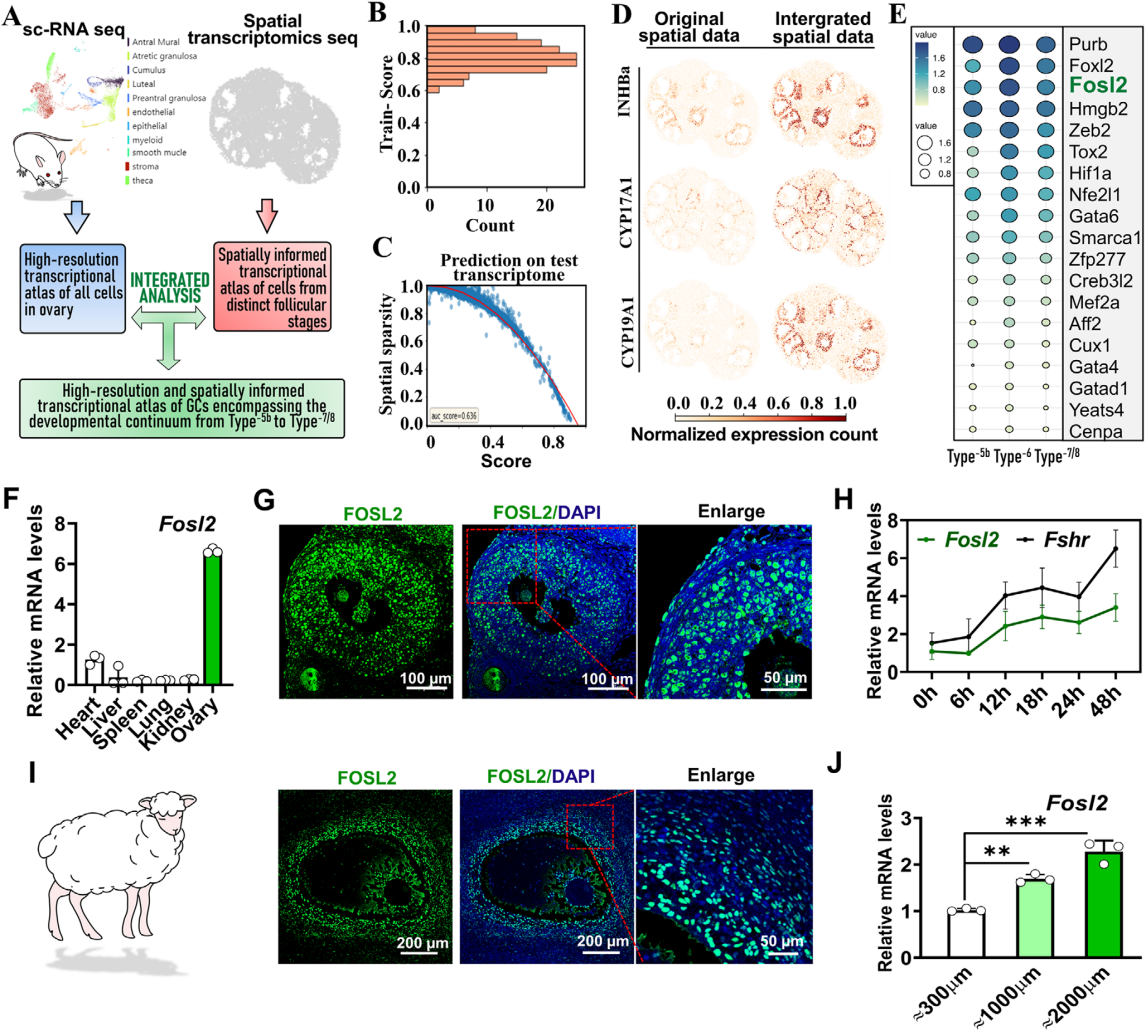
*Fosl2* identified as an FSH‐responsive TF with a dynamic temporal expression pattern that closely mirrored that of the *Fshr*. (A) Schematic representation illustrating the integrative analysis strategy combining scRNA‐seq and spatial transcriptomics. (B) Histogram of gene similarity scores from training data. (C) Scatter plot of gene scores (x‐axis: 0.0–1.0) versus spatial sparsity (y‐axis: 0.0–1.0) from Tangram predictions on integrated transcriptome data. (D) Expression analysis of randomly selected genes showed that the integrated spatial dataset exhibited higher resolution and more distinct spatial localization compared to the original spatial transcriptomics dataset. (E) TFs exhibiting high expression abundances in GCs and upregulated during transition from Type 5 to Type 6–8 as identified through integrative analysis. (F) Tissue expression profile of *Fosl2* across organs. n = 3 mice. (G) Immunofluorescence localization of FOSL2 protein in mouse small antral follicles, with nuclear counterstaining by DAPI. (H) Time‐course expression dynamics of *Fosl2* and *Fshr* in GCs throughout the FSH‐dependent phase. 21‐day‐old mice were injected with 10 IU PMSG to initiate FSH‐dependent follicle maturation, n = 4 GC samples. (I) Immunofluorescence validation of FOSL2 protein localization in sheep small antral follicles, with nuclear visualization via DAPI. (J) qRT‐PCR analysis of *Fosl2* expression in GCs isolated from sheep follicles of different size. n = 3 GC samples. Statistical significance were determined using one‐way ANOVA followed by Tukey's post hoc test, values were mean ± SD. Significant differences were denoted by **P<0.01, ***P<0.001. Shown (F‐J) is a representative result from three independent experiments with similar outcomes.

To identify the FSH‐responsive TFs, we performed single‐cell RNA sequencing (scRNA‐seq) on mouse ovaries. These ovaries, containing follicles at all developmental stages, were harvested from 21‐day‐old mice 48 h after pregnant mare serum gonadotropin (PMSG) injection. This approach facilitated the acquisition of high‐resolution transcriptional landscapes of all GCs in the ovary (Table S1). Subsequently, we conducted a customized analysis of publicly available spatial transcriptomic datasets from mouse ovaries [29], which enabled the generation of spatially informed transcriptomic atlas of GCs from large preantral (Type‐5b), small antral (Type‐6), and large antral follicles (Type‐7/8) (Table S2). By integrating scRNA‐seq data with spatial transcriptomic data (Figure 1A, see Experimental Section), we reconstructed an integrated spatial transcriptional atlas of ovary (Table S3). Following evaluation, the integrated transcriptome dataset demonstrated acceptable quality with gene training scores clustering around 0.8 (Figure 1B) and Area Under the Curve (AUC) values reaching 0.636 (Figure 1C). Expression analysis of randomly selected 15 genes demonstrated that the integrated spatial transcriptomics dataset exhibited higher resolution and more distinct spatial localization compared to the original spatial dataset (Figure 1D; Figure S1). Systematic analysis of this integrated dataset identified 391 TFs exhibiting upregulated expression during the transition from Type‐5b to Type‐7/8 follicles (Table S4). Applying a stringent inclusion criterion of normalized expression count > 0.5 in at least one developmental stage, we identified 19 candidate TFs with robust expression abundances (Figure 1E). Among these, four—*Foxl2*, *Hmgb2*, *Gata6*, and *Gata4—*were excluded due to their previously documented roles in folliculogenesis. From the remaining 15 candidates, *Fosl2* was selected for in‐depth functional analysis based on the four concurrent criteria: (i) it has no previously reported function in folliculogenesis, (ii) qRT‐PCR analysis demonstrated that its expression in the ovary was significantly higher than in other organs (Figure 1F), (iii) Immunofluorescence staining and scRNA‐seq further confirmed its predominant localization within GCs (Figure 1G; Figure S2), (iv) More importantly, its expression is inducible by PMSG stimulation, exhibiting a dynamic temporal expression pattern that closely mirrored that of the *Fshr* (Figure 1H).

Subsequent experiments analyzed *Fosl2*’s expression in sheep follicles, a mono‐ovulatory species. Consistent with findings in mice, *Fosl2* was predominantly expressed in the GCs of sheep follicles (Figure 1I), with a clear positive correlation between expression levels and follicular diameter (Figure 1J). These conserved expression dynamics across polyovulatory and monoovulatory species may support a regulatory role for *Fosl2* in FSH‐dependent follicle maturation.

### 2.2 Knockdown of *Fosl2* Impairs GC Proliferation and Induces Apoptosis

**FIGURE 2:**
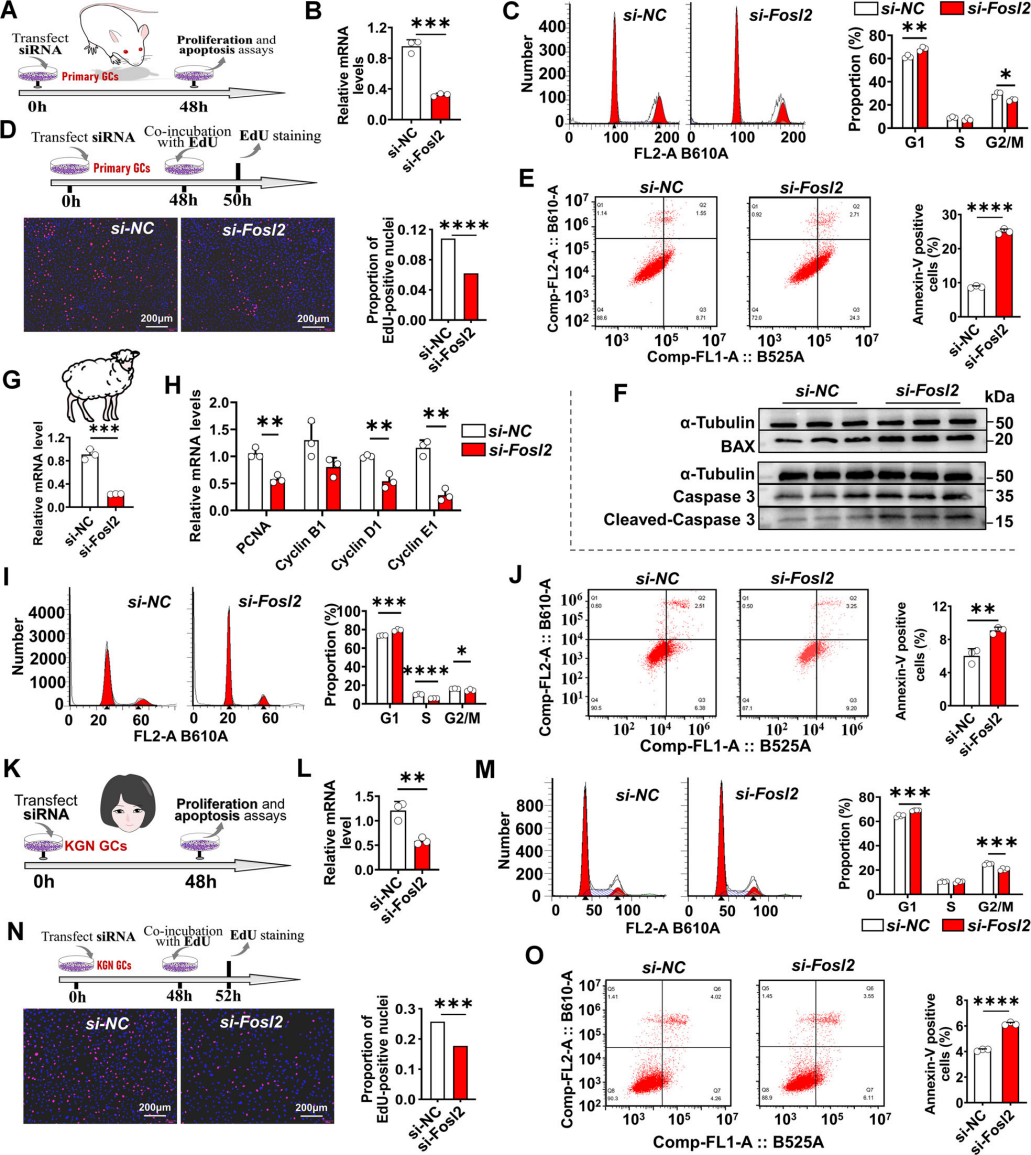
Knockdown of *Fosl2* impairs GC proliferation and induces apoptosis. (A) Schematic of the experimental setup for *Fosl2* knockdown in mouse primary GCs. These cells were harvested from large antral follicles of 21‐day‐old mice 24 h post‐PMSG injection. (B) Knockdown efficiency of *Fosl2* in mouse GCs as determined by qRT‐PCR; n = 3 GC samples. *si‐NC* (scrambled siRNA) served as the negative control. (C) Cell cycle profiling of mouse GCs after *Fosl2* knockdown. Left: representative flow cytometry histograms; right: quantitative analysis of cell cycle phase distribution; n = 3 GC samples. (D) Proliferation of mouse GCs assessed by EdU incorporation assay. Left: representative images of EdU‑positive nuclei (red). Nuclei counterstained with DAPI (blue); right: quantification of EdU‑positive cells. (E) Apoptosis analysis by flow cytometry using Annexin‐V staining. Left: representative flow cytometry plots; right: percentage of Annexin‐V‑positive cells; n = 3 GC samples. (F) Western blot analysis of pro‐apoptotic proteins in mouse GCs after *Fosl2* knockdown; n = 3 GC samples. Original blots were provided in Figure S9. (G) *Fosl2* knockdown in sheep primary GCs. qRT‐PCR validation of knockdown efficiency in GCs isolated from small antral follicles; n = 3 GC samples. (H) qRT‐PCR analysis of proliferation‐related genes in sheep GCs; n = 3 GC samples. (I) Cell cycle analysis of sheep GCs after *Fosl2* knockdown. Left: representative flow cytometry histograms; right: quantification of cell cycle phases; n = 3 GC samples. (J) Apoptosis assessment in sheep GCs by Annexin‐V flow cytometry. Left: representative plots; right: quantitation of Annexin‐V‑positive cells; n = 3 GC samples. (K) Schematic of the experimental setup for *Fosl2* knockdown in human KGN GC line. (L) Knockdown efficiency confirmed by qRT‑PCR; n = 3 GC samples. (M) Cell cycle analysis of KGN cells after *Fosl2* knockdown. Left: representative flow cytometry histograms; right: quantification of cell cycle phases; n = 4 GC samples. (N) EdU incorporation assay in KGN cells. Left: representative images of EdU‑positive nuclei (red). Nuclei counterstained with DAPI (blue); right: quantitation of EdU‑positive cells. (O) Apoptosis measured by Annexin‐V staining in KGN cells. Left: representative flow cytometry plots; right: percentage of Annexin‐V‑positive cells; n = 3 GC samples. Statistical significance was evaluated using a two‐tailed unpaired Student's t‐test or chi‐square test. Data are presented as mean ± SD. Significant differences were denoted by *P<0.05, **P<0.01, ***P<0.001, ****P<0.0001. Shown (B‐J, L‐O) is a representative result from three independent experiments with similar outcomes.

To investigate the functional role of *Fosl2* at cellular level, we first transfected mouse primary GCs with *Fosl2*‐targeting small interfering RNA (siRNA) (Figure 2A). Successful knockdown was confirmed by qRT‐PCR (Figure 2B). Cell cycle analysis via flow cytometry revealed that *Fosl2* depletion resulted in dysregulation, characterized by accumulation of cells in the G1 phase and a concomitant decrease in the G2/M phase population (Figure 2C). This anti‐proliferative effect was further corroborated by EdU incorporation assay, which showed a marked reduction in DNA synthesis in *Fosl2*‐silenced GCs compared to controls (Figure 2D). In addition to impairing proliferation, *Fosl2* knockdown promoted apoptosis. Flow cytometric analysis using Annexin‐V staining indicated a significant increase in the proportion of cells in early apoptosis (Figure 2E). Consistent with this, western blot analysis revealed elevated levels of the pro‐apoptotic proteins (BAX and cleaved Caspase‐3) in *Fosl2*‐ knockdown GCs (Figure 2F), further confirming the activation of apoptotic pathways.

We next assessed whether the function of *Fosl2* is conserved across species. In sheep primary GCs, *Fosl2* knockdown led to significant downregulation of proliferation markers, including *PCNA*, *Cyclin D1*, and *Cyclin E1* (Figure 2G,H). Cell cycle profiling displayed G1 phase accumulation and a reduced proportion of cells in S and G2/M phase in the knockdown group (Figure 2I). It also resulted in a significant increase in Annexin‐V‐positive cells (Figure 2J), mirroring the pro‐apoptotic effect observed in mouse GCs.

To extend these findings to a human model, we performed *Fosl2* knockdown in the human KGN GC line (Figure 2K,L). Similarly, cell cycle profiling showed G1 phase accumulation and a reduced proportion of cells in G2/M phase upon *Fosl2* knockdown (Figure 2M). EdU incorporation was significantly diminished in *Fosl2*‐knockdown KGN GCs (Figure 2N), and apoptosis was enhanced, as evidenced by increased Annexin‐V staining (Figure 2O).

### 2.3 Knockdown of *Fosl2* Impairs FSH‐Dependent Follicle Maturation In Vitro

**FIGURE 3:**
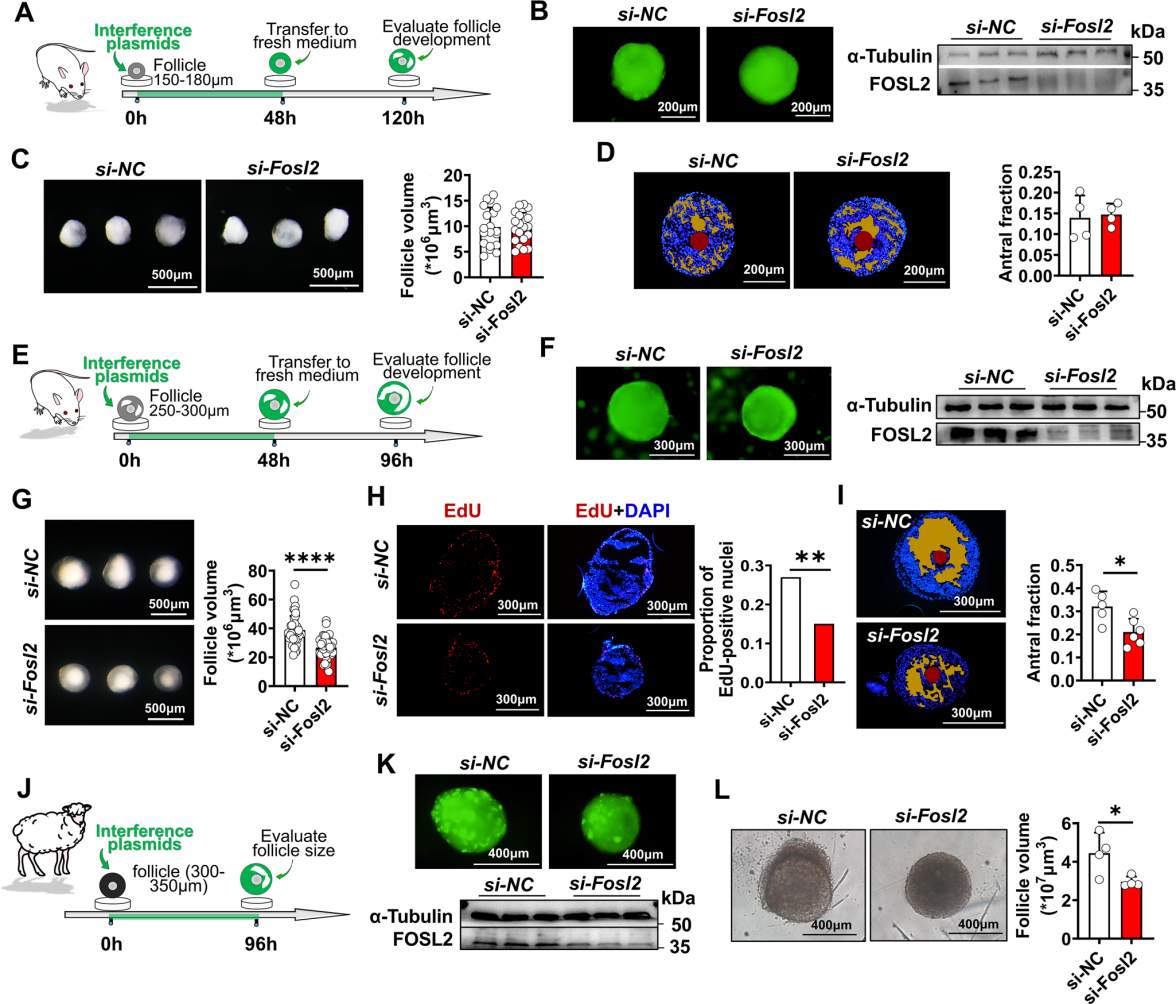
Knockdown of *Fosl2* impairs FSH‐dependent follicle maturation in vitro. (A) Schematic illustration of the experimental design for *Fosl2* knockdown in mouse FSH‐independent follicles. These follicles were isolated from the ovaries of 15‐day‐old mice. (B) Evaluation of *Fosl2* knockdown efficiency by western blot; n = 3 follicular samples. Green fluorescence indicates successful plasmid transfection in follicles. Original blots were provided in Figure S9. (C) Analysis of follicle volume changes following *Fosl2* knockdown; n = 19 follicles. (D) Change in follicular antral fraction following *Fosl2* knockdown; n = 4 follicles. Nuclei counterstained with DAPI. Yellow area represents the follicular antrum. Red area represents the oocyte. (E) Schematic illustration of the experimental design for *Fosl2* knockdown in mouse FSH‐dependent follicles. These follicles were isolated from the ovaries of 19‐day‐old mice. (F) Evaluation of *Fosl2* knockdown efficiency by western blot; n = 3 follicular samples. Original blots were provided in Figure S9. (G) Follicle volume changes following *Fosl2* knockdown; n = 42 follicles (*si‐NC*), n = 37 follicles (si‐*Fosl2*). (H) Follicular cell proliferation analysis using the EdU incorporation assay. Left: representative images of EdU‑positive nuclei (red). Nuclei counterstained with DAPI (blue); right: quantification of EdU‐positive nuclei; n = 7 (*si‐NC)* and n = 6 (si‐*Fosl2*) follicles. (I) Changes in the follicular antral fraction post‐*Fosl2* knockdown; n = 5 (*si‐N*C) and n = 6 (si‐*Fosl2*) follicles. (J) Schematic illustration of the experimental design for *Fosl2* knockdown in sheep FSH‐dependent follicles. (K) Evaluation of *Fosl2* knockdown efficiency by western blot; n = 3 follicular samples. Original blots were provided in Figure S9. Green fluorescence indicates successful transcription of interfering plasmids in sheep follicles. (L) Analysis of sheep follicle volume following *Fosl2* knockdown; n = 4 follicles. Statistical significance was determined using two‐tailed unpaired Student's t‐test or chi‐square test, values were mean ± SD. Significant differences were denoted by *P<0.05, **P<0.01, ****P<0.0001. Shown (B‐D, F‐I, K and L) is a representative result from three independent experiments with similar outcomes.

To elucidate the functional relevance of *Fosl2* during follicle maturation, we performed stage‐specific lentiviral shRNA‐mediated knockdown of *Fosl2* in cultured follicles, aiming to dissect its role at distinct developmental stages (Figure 3A,B). In small preantral follicles, targeted knockdown of *Fosl2* over a 5‐day ex vivo culture period did not result in any overt morphological or functional abnormalities. Quantitative assessments revealed no significant differences in follicular size (Figure 3C), the proportion of EdU‐positive GCs (Figure S3A), or the antral fraction (Figure 3D) between the knockdown and control groups. These findings suggest that *Fosl2* is dispensable for FSH‐independent follicle maturation and antrum emergence.

In contrast, *Fosl2* knockdown in small antral follicles led to notable developmental impairments. After 4 days of culture, the knockdown group exhibited a reduced follicular size (Figure 3E–G), downregulated expression of proliferation‐related genes (Figure S3B), and a lower proportion of EdU‐positive GCs compared to controls (Figure 3H). Additionally, knockdown follicles demonstrated defective antrum expansion, characterized by a 34.5% reduction in the antral fraction (Figure 3I). These results indicate that *Fosl2* is specifically involved in regulating FSH‐dependent follicle maturation.

Consistent with the phenotype observed in mouse follicles, FSH ‐ dependent follicles in sheep with *Fosl2*‐knockdown (Figure 3J) also displayed reduced size following 4‐day culture relative to controls (Figure 3K,L), highlighting a conserved role for *Fosl2* in mammalian follicle maturation across species.

### 2.4 GC‐Specific *Fosl2* Knockout Blocks FSH‐Dependent Follicle Maturation and Causes Infertility

**FIGURE 4:**
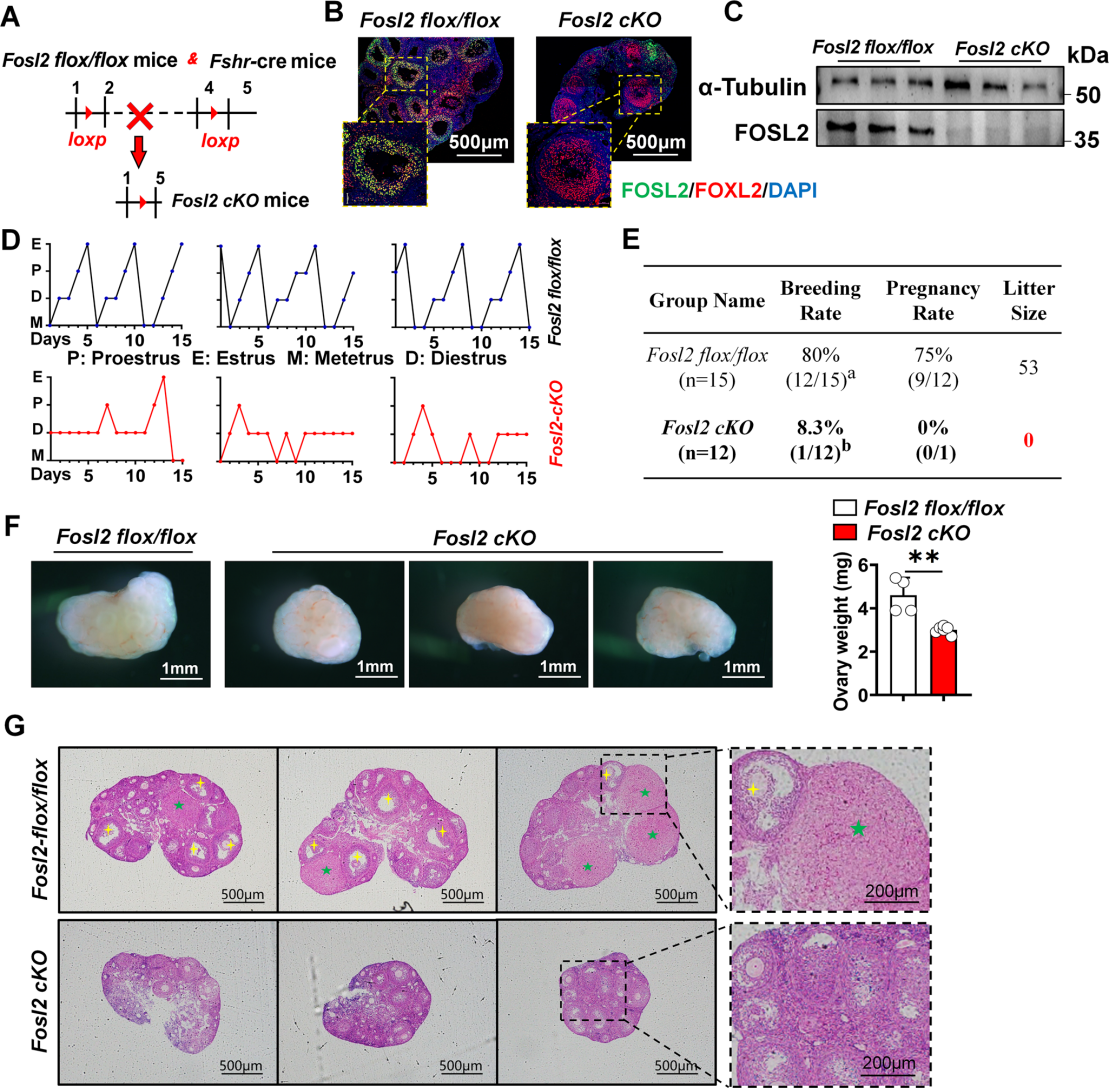
GC‐specific *Fosl2* knockout blocks FSH‐dependent follicle maturation and causes infertility. (A) Schematic representation of the conditional knockout of *Fosl2* in GCs of C57BL/6J mouse. (B) Immunofluorescence analysis revealed the successful ablation of *Fosl2* expression in GCs of large preantral follicles. Blue: DAPI (nuclear stain), Green: FOSL2, Red: FOXL2. FOXL2 is abused as lineage marker for GCs. (C) Western blot analysis confirming the successful deletion of *Fosl2* in GCs; n = 3 GC samples. GCs were isolated from ovaries of 21‐day‐old mice 48 h post‐PMSG injection. Original blots were provided in Figure S9. (D) Evaluation of reproductive cycle alterations following *Fosl2* knockout, n = 10 mice. (E) Assessment of reproductive capacity in *Fosl2 cKO* mice. (F) Analysis of ovarian weight. Left: representative images of ovaries; right: quantification of ovarian weight; n = 4 (*Fosl2 flox/flox*) and 6 (*Fosl2 cKO*) ovaries. Ovaries were harvested from 21‐day‐old mice 48 h post‐PMSG injection. (G) Morphological examination of ovaries via HE staining. Ovaries were harvested from 2‐month‐old mice. Yellow stars indicate large antral follicles, while green stars indicate corpus lutea. Statistical significance was determined using two‐tailed unpaired Student's t‐test, values were mean ± SD. Significant differences were denoted by **P<0.01. Different letters (a, b) indicate a significant difference. Shown (F and G) is a representative result from two independent experiments with similar outcomes.

To further investigate the functional necessity of *Fosl2* in FSH‐dependent follicle maturation, we generated GC‐specific *Fosl2* conditional knockout (*Fosl2 cKO*) mice using an *Fshr‐Cre* driver system (Figure 4A). Immunofluorescence staining (Figure 4B) and western blot (Figure 4C) assays confirmed the efficient and specific ablation of *Fosl2* in GCs of large preantral follicles. Phenotypic analysis of *Fosl2 cKO* females revealed profound reproductive defects. Specifically, longitudinal observation across three consecutive reproductive cycles showed complete cessation of reproductive cycle in all ten *cKO* mice involved in this assessment (Figure 4D). During a 15‐day mating trial, all twelve *cKO* females failed to give birth to offspring (Figure 4E).

Ovarian phenotyping performed 48 h after PMSG administration revealed substantially reduced ovarian weights in *cKO* mice compared to control *Fosl2 flox/flox* littermates (Figure 4F). Furthermore, GCs isolated from *cKO* ovaries displayed marked downregulation of proliferation‐associated genes (*PCNA*, *Ki67* and *Cyclin E1*) and concomitant upregulation of pro‐apoptotic proteins (cleaved Caspase‐3, BAX) relative to *Fosl2 flox/flox* controls (Figure S4). The ovaries of 2‐month‐old mice were also isolated for Hematoxylin and eosin (HE) staining, it was revealed that *Fosl2 flox/flox* ovaries contained large antral follicles and corpora lutea, indicating normal ovarian function, whereas *Fosl2 cKO* ovaries exhibited follicles that developed only to the small antral stage, with no corpora lutea observed (Figure 4G). These morphological and molecular alterations provide compelling evidence that disruption of *Fosl2* function impedes FSH‐dependent follicle maturation, thereby establishing a mechanistic link between the observed reproductive phenotypes (anestrus and infertility) and impaired follicle maturation. These findings establish *Fosl2* as an indispensable TF required for FSH‐dependent follicle maturation.

### 2.5 *Fosl2* Amplifies FSH/FSHR Signaling via Transcriptional Upregulation of *Fshr* and Stimulation of Estrogen Production

**FIGURE 5:**
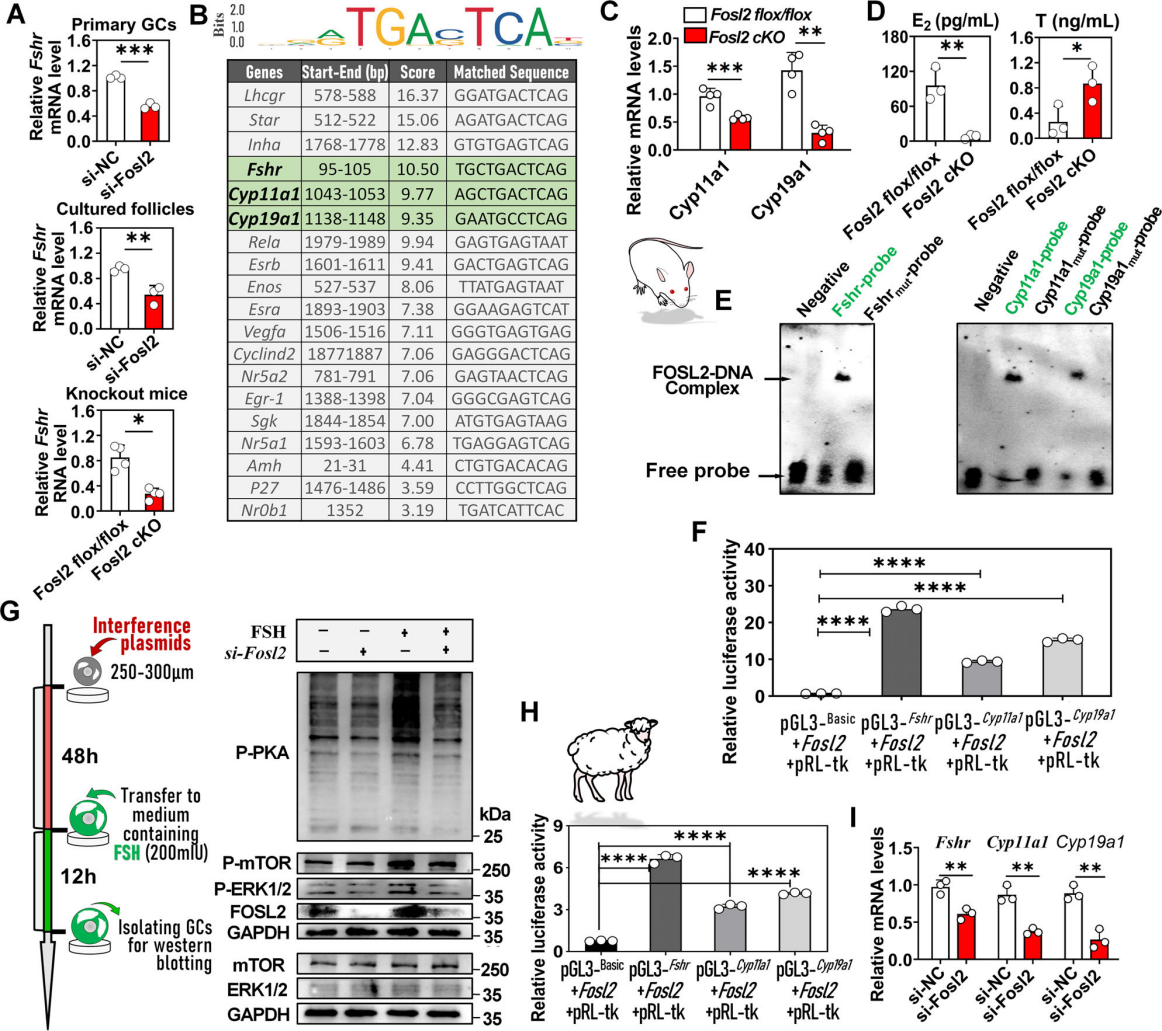
*Fosl2* amplifies FSH/FSHR signaling via transcriptional upregulation of *Fshr* and stimulation of estrogen production (A) qRT‐PCR analysis of *Fshr* expression following disruption of *Fosl2*. n = 3 (primary GCs, cultured follicles) and n = 4 (*cKO* mice). The *Fosl2*‐knockout GCs were harvested from 21‐day‐old mice 48 h post‐PMSG injection. (B) Bioinformatic prediction of *Fosl2* target genes. (C) qRT‐PCR analysis of *Cyp11a1* and *Cyp19a1* expression levels following *Fosl2* knockout. GCs were collected as in (A); n = 4 GC samples. (D) Changes in levels of serum estradiol (E2) and testosterone (T) following *Fosl2* knockout. Serum samples were harvested from 21‐day‐old mice 48 h after PMSG injection (n = 3 mice). (E) EMSA validating direct binding of FOSL2 protein to promoter regions of *Fshr*, *Cyp11a1* and *Cyp19a1*. (F) Dual‐luciferase reporter assay assessing transcriptional activation of *Fshr*, *Cyp11a1*, and *Cyp19a1* promoters by *Fosl2* in primary mouse GCs, n = 3 GC samples. (G) Effect of *Fosl2* knockdown on canonical FSH‐downstream pathways. Left: schematic of experimental design; right: western blot analysis. Original blots were provided in Figure S9. (H) Dual‐luciferase reporter assay confirming *Fosl2*‐mediated transactivation of *Fshr*, *Cyp11a1*, and *Cyp19a1* promoters in sheep primary GCs, n = 3 GC samples. (I) qRT‐PCR analysis of *Fshr*, *Cyp11a1*, and *Cyp19a1* expression in sheep GCs isolated from small antral follicles following *Fosl2* knockdown; n = 3 GC samples. Statistical significance was determined using two‐tailed unpaired Student's t‐test, values were mean ± SD. Significant differences were denoted by*P<0.05, **P<0.01, ***P<0.001, ****P<0.0001. Shown is a representative result from three (E, G) and two (A, C, D, F, H and I) independent experiments with similar outcomes.

To investigate whether *Fosl2* amplifies FSH/FSHR signaling via upregulation of *Fshr*, thus regulating FSH‐dependent follicle maturation, we first examined the impact of *Fosl2* loss on *Fshr* expression in GCs. qRT‐PCR analysis showed that *Fshr* mRNA levels were significantly reduced following *Fosl2* knockdown in vitro (in both cellular and follicular models) as well as after in vivo knockout (Figure 5A).

We next employed integrated bioinformatic, biochemical, and functional approaches to confirm direct transcriptional regulation of *Fshr* by *Fosl2*. Conserved FOSL2 protein‐binding sites were predicted within the *Fshr* promoter using the JASPAR and FIMO databases. In addition, FOSL2 protein‐binding sites were also identified in the promoters of *Cyp11a1* and *Cyp19a1*, key genes regulating estrogen biosynthesis—a steroid hormone known to in amplifies FSH/FSHR signaling (Figure 5B). Consistent with the reduction in *Fshr* (Figure 5A), disruption of *Fosl2* led to significant downregulation of *Cyp11a1* and *Cyp19a1* expression in GCs (Figure 5C) and decreased estrogen levels in serum and culture medium (Figure 5D; Figure S5), compared to *Fosl2 flox/flox* controls. Notably, the serum testosterone levels were significantly elevated in *cKO* mice compared to controls (Figure 5D). While impaired aromatization is a likely explanation for this elevation, alternative mechanisms such as altered theca cell function or paracrine signaling could also contribute. Validation through electrophoretic mobility shift assays (EMSA) confirmed direct binding of recombinant FOSL2 protein to promoter regions of *Fshr*, *Cyp11a1* and *Cyp19a1* (Figure 5E). Furthermore, dual‐luciferase reporter assays demonstrated that FOSL2 protein significantly enhances the transcriptional activity of all three promoters (Figure 5F).

We further evaluated the effect of *Fosl2* on key FSH‐downstream signaling pathways—cAMP‐PKA, ERK and mTOR—in cultured follicles. Western blot analysis revealed that FSH stimulation significantly enhanced the phosphorylation of PKA, ERK and mTOR in GCs. In contrast, knockdown of *Fosl2* abolished the FSH‐induced upregulation of phosphorylation in all three proteins (Figure 5G). This result supports a role for *Fosl2* in amplifying FSHR‐mediated signal transduction.

Collectively, these results indicate that *Fosl2* reinforces *Fshr* expression and stimulates estrogen production at the transcriptional level, thereby amplifying FSH/FSHR signaling and facilitating FSH‐dependent follicle maturation. The conservation of this regulatory mechanism was confirmed in sheep GCs, where dual‐luciferase assays showed *Fosl2*‐dependent transactivation of the *Fsh*r, *Cyp11a1*, and *Cyp19a1* promoters (Figure 5H). Additionally, *Fosl2* knockdown in both sheep and human GCs led to significant downregulation of these genes (Figure 5I; Figure S6).

### 2.6 FSH/FSHR Signaling Activates *Fosl2* Transcription via the cAMP‐PKA‐CREB

**FIGURE 6:**
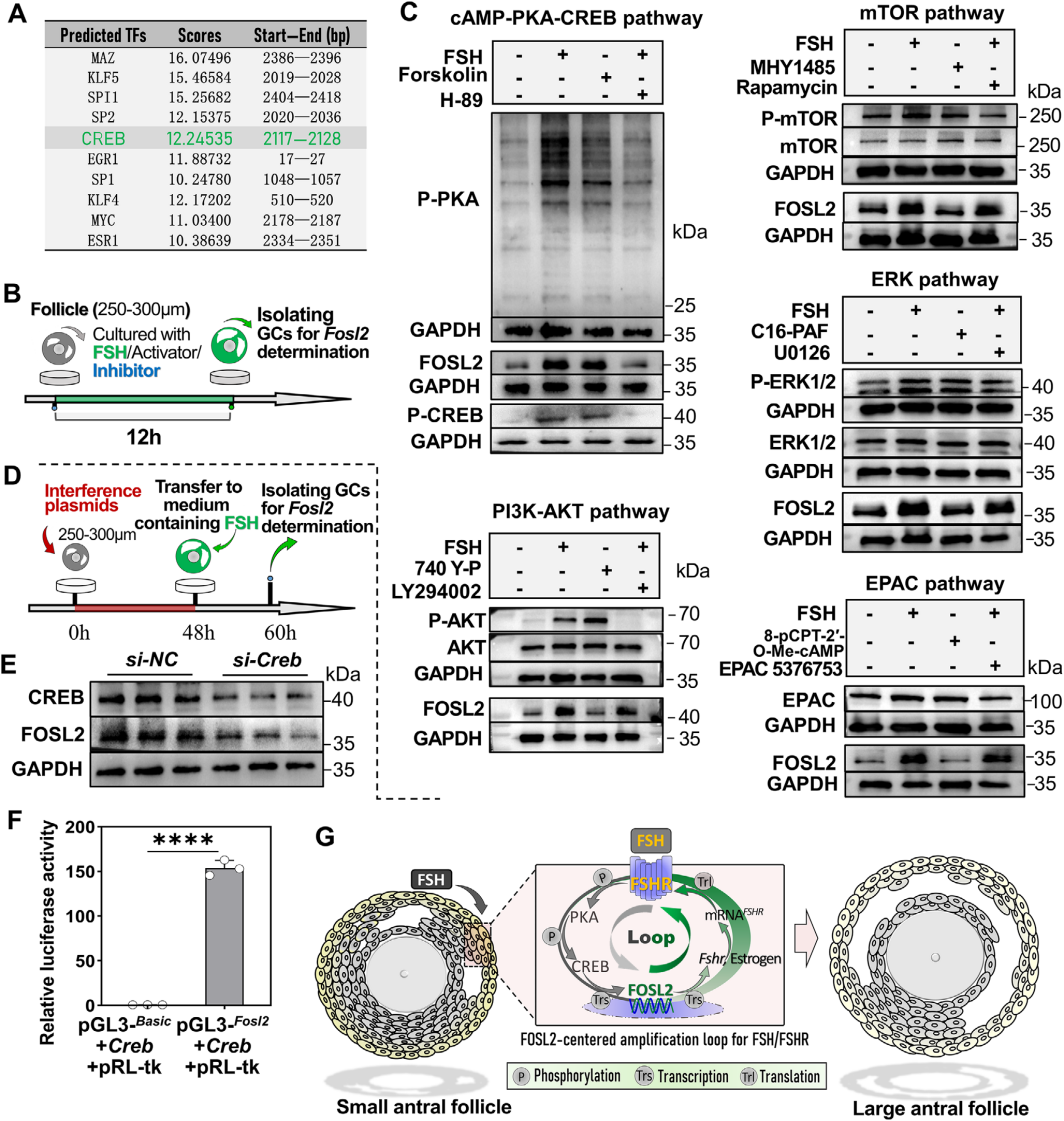
FSH/FSHR signaling activates *Fosl2* transcription via the cAMP‐PKA‐CREB. (A) Prediction of TFs binding to *Fosl2*’s promoter using JASPAR database. (B) Schematic representation illustrating the experimental design for panel C. (C) Changes in FOSL2 protein contents in mouse GCs following activation or inhibition of the FSH‐downstream pathways. Adenylate cyclase activator: forskolin (20 µM), PKA inhibitor: H‐89 (50 µM);PI3K activator: 740 Y‐P (30 µM), PI3K inhibitor: LY294002 (100 µM); mTOR activator: MHY1485 (5 µM), mTOR inhibitor: Rapamycin (1 µM); ERK activator: C16‐PAF (10 µM), ERK inhibitor: U0126 (40 µM); EPAC activator: 8‐pCPT‐2’‐O‐Me‐cAMP (2 µM), EPAC inhibitor: EPAC 5376753 (10 µM). Original blots were provided in Figure S10. (D) Schematic representation illustrating the experimental design for panel E. (E) Changes of FOSL2 protein contents in mouse GCs following *Creb* knockdown. Original blots were provided in Figure S10. (F) Dual‐luciferase reporter assay confirming CREB‐mediated transactivation of the *Fosl2* promoter. Experiments were conducted in primary mouse GCs, n = 3 GC samples. (G) A diagram depicting the *Fosl2*‐centered positive feedback loop that amplifies FSH/FSHR signaling. During FSH‐dependent follicle maturation, FSH induces *Fosl2* expression via the cAMP‐PKA‐CREB cascade. FOSL2 in turn binds the promoters of *Fshr* and estrogen‐biosynthesis genes to enhance their transcription, thereby increasing *Fshr* mRNA level and amplifying FSH/FSHR signaling. Statistical significance was determined using two‐tailed unpaired Student's t‐test, values were mean ± SD. Significant differences were denoted by ****P<0.0001. Shown is a representative result from three (C, E) or two (F) independent experiments with similar outcomes.

To further elucidate the mechanism by which FSH/FSHR activates *Fosl2* transcription, we employed JASPAR database to predict potential TFs binding to the promoters of *Fosl2*. As shown in Figure 6A, among ten predicted TFs with the high binding affinity scores, CREB—a key downstream TF of the PKA kinase cascade—was identified. Given that FSHR is a G protein‐coupled receptor capable of activating target gene transcription through the PKA kinase cascade, we hypothesized that the cAMP‐PKA‐CREB cascade mediates *Fosl2* transcription. To test this, we conducted experiments using a follicle culture system (Figure 6B). Treatment with forskolin, an adenylate cyclase activator, led to significant increase of P‐PKA, P‐CREB and FOSL2 in the absence of exogenous FSH. In contrast, introduce of H‐89, a PKA inhibitor, effectively blocked FSH‐induced increases in P‐CREB and FOSL2, confirming the involvement of PKA activity in this regulatory process. Furthermore, activation of other classical FSH/FSHR downstream pathways—including PI3K‐AKT, ERK, mTOR, and EPAC—did not elevate FOSL2 protein levels in GCs; similarly, inhibition of these pathways failed to suppress the FSH‐induced increase in FOSL2 protein level (Figure 6C).

To validate the direct role of CREB in regulating *Fosl2* transcription, we performed *Creb* knockdown in cultured follicles (Figure 6D). Compared to the controls, *Creb* knockdown significantly reduced FOSL2 protein levels (Figure 6E). Lastly, a dual‐luciferase reporter assay demonstrated that CREB protein markedly enhances the transcriptional activity of the *Fosl2* promoter (Figure 6F), highlighting its essential role as a transcriptional activator of *Fosl2*. Altogether, these findings confirm that FSH/FSHR signaling activates *Fosl2* transcription via the cAMP‐PKA‐CREB cascade.

## 3 Discussion

FSH/FSHR signaling orchestrates folliculogenesis, with its amplification being essential for successful FSH‐dependent follicle maturation. While mechanisms underlying this amplification remain incompletely resolved, emerging evidence implicates a multilayered regulatory network. Systemic factors (e.g., activin, myostatin) enhance pituitary FSH secretion [30, 31], whereas local GC‐intrinsic pathways—including estrogen signaling, TGF‐*β*/Smad, Wnt/*β*‐catenin, IGF1, and NGF—upregulate *Fshr* expression, further augmenting FSH/FSHR signaling [26, 32, 33, 34, 35].

Here, we identify *Fosl2* as a previously unrecognized regulator of FSH/FSHR amplification. Through integrated in vitro and in vivo analyses, we demonstrate that FSH/FSHR activation upregulates *Fosl2* expression via the cAMP‐PKA‐CREB cascade. FOSL2 protein in turn binds to the *Fshr* promoters, enhancing its transcriptional activity. This action immediately elevates *Fshr* mRNA level, thereby establishing a positive feedback loop that amplifies FSH/FSHR signaling. Functionally, this *Fosl2*‐mediated loop acts as a molecular switch essential for FSH‐dependent follicle maturation (Figure 6G). Additionally, our study demonstrates that FOSL2 also directly transactivates *Cyp11a1* and *Cyp19a1* (Figure 5C), key enzymes involved in estrogen biosynthesis. Disruption of *Fosl2* at cellular, follicular and organismal levels uniformly impaired estrogen synthesis (Figure 5D; Figures S5 and S6). Since estrogen plays a significant role in augmenting *Fshr* transcription [26], *Fosl2* likely amplifies FSH/FSHR via dual complementary mechanisms: direct *Fshr* transactivation and indirect amplification via regulating estrogen production.

Notably, FOSL2 specifically potentiates the FSH‐induced upregulation of *Fshr* but without influencing its basal expression. This is robustly supported by experimental evidence (Figure S7): under FSH stimulation, follicles exhibited accelerated growth concomitant with a marked increase in *Fshr* expression. Ablating *Fosl2* expression significantly impaired both FSH‐promoted follicle growth and the concomitant upregulation of *Fshr*. In contrast, in the absence of FSH, follicular development remained slow, baseline *Fshr* mRNA level was unchanged, and *Fosl2* knockdown had no discernible effect on either follicle growth or basal *Fshr* expression. In addition, this study does not test whether restoring *Fshr* expression or downstream estrogen signaling alone can bypass the *Fosl2* requirement. Therefore, while *Fosl2* deletion completely blocks FSH‐dependent follicle maturation—largely attributable to the downregulation of *Fshr*, *Cyp11a1* and *Cyp19a1—*we recognize that this may not be the exclusive cause. As a canonical TF, *Fosl2* likely regulates additional transcriptional programs contributing to the phenotype.

FOSL2 protein is a pleiotropic TF activated by diverse stimuli, including hormones, growth factors, cytokines, and extracellular matrix signals [36, 37]. Global *Fosl2* knockout causes neonatal lethality [38], while tissue‐specific models have revealed its essential roles in osteogenesis [39, 40], lipid metabolism [41], lung function [42, 43, 44], immune responses [45, 46], and oncogenesis [47, 48, 49, 50]. Nevertheless, the function of *Fosl2* in reproductive physiology remains poorly defined. In this study, we establish that *Fosl2* amplifies FSH/FSHR signaling through transcriptional upregulation of *Fshr* and potentiation of estrogen synthesis, thereby specifically enabling FSH‐dependent follicle maturation. This finding addresses a critical knowledge gap regarding the reproductive functions of *Fosl2*.

Following the posting of this finding on bioRxiv [51], we take note of a recent study explored *Fosl2* in follicle maturation [52]. In that work, Zhang et al. utilized *Cyp19a1‐Cre* to achieve targeted deletion of *Fosl2* in GCs. Unlike *Fshr‐Cre*, which mediates deletion beginning at the large preantral follicular stage, *Cyp19a1‐Cre* induces deletion specifically starting from the antral follicular stage. They reported that *Fosl2* loss at this late stage did not compromise fertility in mice. The knockout mice largely maintained normal ovarian function, with only a delayed subfertility phenotype emerging in aging females. They thus concluded that GC‐specific deletion of *Fosl2* disrupts GC cellularity, leading to subfertility associated with reproductive aging. In light of this, our study does more than just complement their work; it provides the essential context necessary for the correct interpretation of *Fosl2*’s in vivo function. The mild, aging‐associated subfertility reported by Zhang et al. can now be understood as a secondary consequence of losing *Fosl2* during later, periovulatory stage, while our research reveals the profound phenotypic defect caused by earlier *Fosl2* loss: a failure to successfully complete the FSH‐dependent follicle maturation. Therefore, a complete and accurate understanding of *Fosl2* in reproduction requires integrating the insights from both studies, with ours defining the critical functional window of *Fosl2* occurs during the small antral‐to‐large antral transition, rather than at the periovulatory stage.

Having validated the conserved role of *Fosl2* in FSH‑dependent follicle maturation in mice and sheep, we expanded our study to examine its function in human ovarian biology and related reproductive disorders. Interrogation of public datasets from patients with premature ovarian insufficiency (POI) [53] revealed decreased *Fosl2* expression (Figure S8), and its knockdown in human GCs reduced cell viability (Figure 2M–O), suggesting a possible link between altered *Fosl2* levels and ovarian pathology. However, direct functional assessment of *Fosl2* in human chorionic gonadotropin (hCG)‐untreated primary GCs or intact follicles was precluded by practical limitations. Instead, loss‑of‑function experiments were conducted using the human tumor‑derived GC line KGN. Consequently, the observed correlations from POI data and KGN models do not demonstrate causality for *Fosl2* in human folliculogenesis or POI. Although the KGN system provides useful insights, these results warrant cautious interpretation and underscore the need for further studies to define the precise role of *Fosl2* in human folliculogenesis and ovarian disease etiology.

In this study, the full scope of *Fosl2* target genes remains undefined. Although we establish its direct regulation of *Fshr*, *Cyp11a1*, and *Cyp19a1*, these targets may not fully account for all observed phenotypic outcomes. We initially considered ChIP‐seq but found no commercially available FOSL2 antibody suitable for robust chromatin immunoprecipitation in our hands. RNA‐seq following *Fosl2* perturbation was also considered, however, after discovering that *Fosl2* potentiates FSH signaling, we reasoned that such an approach would be inconclusive. Given that FSH regulates a broad transcriptional network, RNA‑seq data—in the absence of complementary ChIP‑seq—would be unable to discriminate between direct *Fosl2* targets and secondary effects mediated through enhanced FSH signaling. As an alternative, we adopted a candidate‐gene strategy, validating *Fosl2* binding to a limited set of promoters via bioinformatic prediction, EMSA, and luciferase reporter assays. While this approach confirmed specific interactions, it inevitably results in an underestimation of the full repertoire of *Fosl2* target genes. A further limitation is the absence of follicle‐level rescue experiments. We attempted but could not develop a reliable method for sustained *Fosl2* overexpression in cultured follicles to test functional rescue. Consequently, it should be acknowledged that the absence of rescue data may limit the physiological conclusiveness regarding sufficiency.

In summary, this study elucidates a *Fosl2*‐driven feedback loop essential for amplifying FSH/FSHR. Disruption of this loop complete blocks FSH‐dependent follicle maturation, underscoring *Fosl2*’s indispensable role in reproduction.

## 4 Experimental Section

### 4.1 Experimental Design

This study followed a stepwise and logical strategy: starting from omics‐based prediction, progressing to cellular assays, advancing to follicle culture models, and ultimately validating findings in a whole‐organism context using a conditional knockout mouse model. This sequential approach ensured that each stage of validation informed the next, thereby minimizing reliance on any single methodological platform. By integrating single‐cell and spatial transcriptomic analyses, candidate TFs exhibiting upregulation in GCs during folliculogenesis were identified. Subsequently, qRT‐PCR was employed to screen for FSH‐responsive TFs among the candidates. From these, TF was selected for in‐depth functional analysis based on the following concurrent criteria: (i) it has no previously reported function in folliculogenesis, (ii) it exhibits high expression abundance in GCs, (iii) its expression is inducible by PMSG stimulation, and (iv) its expression dynamics closely mirror those of *Fshr* during the FSH‐dependent phase.

Following the identification of *Fosl2* as the research subject, knockdown experiments were conducted in GCs derived from mouse, sheep and human to examine its role in regulating proliferation, apoptosis, and estrogen synthesis. To investigate the functional relevance of *Fosl2* in a more physiologically relevant background, *Fosl2* was knocked down in cultured mouse and sheep follicles to determine whether its knockdown selectively disrupts FSH‐dependent follicle maturation and estrogen secretion. In parallel, GC‐specific *Fosl2* knockout mice were generated to evaluate the in vivo effects of *Fosl2* deficiency on FSH‐dependent follicle maturation, estrous cyclicity, estrogen production, and reproductive capacity.

To explore the potential mechanism through which *Fosl2* modulates FSH‐dependent follicle maturation—specifically its role in amplifying FSH/FSHR signaling—bioinformatic prediction was performed to evaluate FOSL2 protein's binding affinity to the *Fshr* promoter. This prediction was experimentally validated using EMSA and luciferase reporter assays. Furthermore, *Fosl2* knockdown was carried out in cultured FSH‐dependent follicles, followed by western blot to assess the resulting changes in the activity of four downstream pathways of FSH/FSHR: Akt, cAMP‐PKA, mTOR, ERK.

Finally, a combination of western blot, RNA interference, and luciferase reporter assays was employed in a follicle culture system to elucidate the downstream signaling cascade through which FSH/FSHR upregulates *Fosl2* transcription.

The time points for sampling were selected based on the established dynamics of PMSG‐induced follicle development from our previous study [22]. GC proliferation peaks at 24 h post‐PMSG, hence its use for proliferation assays. Expression of *Fshr*, *Cyp11a1*, and *Cyp19a1* peaks at 48 h, making this time point appropriate for their analysis and for assessing overall follicle maturation.

### 4.2 Animals

Kunming mice were purchased from the Center for Animal Testing of Huazhong Agricultural University (Wuhan, China). *Fosl2 flox/flox* C57BL/6J mice were purchased from GemPharmatech Co., Ltd. (Nanjing, China). Following the mating of *Fosl2 flox/flox* mice with *Fshr‐Cre* mice, specific deletion of exons 2 to 4 of *Fosl2* in GCs can be achieved. All experiments in mice used the Kunming strain, with the exception of knockout studies, which were conducted on the C57BL/6J strain. Mice were reared in a specific pathogen free laboratory animal house, at a constant temperature of 22 ± 2°C, being allowed to access food and water ad libitum with 12 h light‐dark cycles. Sheep ovaries were purchased from slaughterhouses. Prior approval from the Institutional Animal Ethics Committee of Huazhong Agricultural University was obtained, with the approved protocol number being HZAUMO‐2022‐0181 (mouse); HZAUSH‐2025‐0002 (sheep).

### 4.3 Reproductive Cycle Determination

Vaginal smears were performed to determine the reproductive cycle stage in mice. Briefly, 50 µL of physiological saline was introduced into the vaginal canal and gently aspirated three times to obtain cellular samples. The lavage fluid was then transferred onto glass slides and air‐dried. The samples were stained by HE staining kit (Servicebio, China). After washing with distilled water, the slides were air‐dried completely and examined under a light microscope. The reproductive cycle stage (proestrus, estrus, metestrus, or diestrus) was determined based on the predominant cell types observed: nucleated epithelial cells, cornified epithelial cells, leukocytes, or their combinations [54].

### 4.4 Integrative Analysis of scRNA‐seq and Spatial Transcriptomics

While scRNA‐seq provides high‐resolution transcriptional profiles of GCs, it exhibits limited accuracy in distinguishing GCs across distinct follicular stages. Conversely, spatial transcriptomics precisely resolves follicular staging but lacks the depth of scRNA‐seq. To address these limitations, we integrated both modalities to achieve high‐resolution and spatially informed transcriptional atlas of GCs across follicle maturation. Ovaries from PMSG (Ningbo Sansheng Biological Technology, China)‐injected mice (48 h post‐injection), containing follicles at all developmental stages, were collected for scRNA‐seq (10× Genomics platform) by Yingzi Gene (Wuhan, China). Cell clustering was performed using a graph‐based approach, which involved constructing a sparse nearest‐neighbor graph followed by Louvain Modularity Optimization for community detection [55]. Differential gene expression analysis between cell clusters was conducted using the Seurat Bimod test, with significance thresholds set at a false discovery rate (FDR) ≤ 0.05 and an absolute log_2_ fold change ≥ 1.5. Spatial transcriptomic data from mouse ovaries (48 h post‐PMSG injection), originally generated by Mantri et al., were retrieved from the Gene Expression Omnibus (GEO) database (Accession: GSE240271), with processed metadata and cell annotation files obtained from the associated GitHub repository (https://github.com/madhavmantri/mouse_ovulation).

We integrated the scRNA‐seq and spatial transcriptomic data following an established methodology [56, 57]. No batch correction or sensitivity analysis was performed prior to Tangram mapping. This decision was based on the high comparability of the datasets: both scRNA‐seq and spatial transcriptomics samples were from 21‐day‐old prepubertal mice, collected 48 h after PMSG stimulation. The workflow of integrative analysis began with data preprocessing, where total Unique Molecular Identifier (UMI) counts in scRNA‐seq data were normalized using *scanpy.pp.normalize_total* to eliminate variability in sequencing depth. Spatial mapping was then performed using a deep learning model (*tangram.map_cells_to_space*) implemented in PyTorch, trained on 189 ovarian marker genes [58]. The high‐resolution capability of this approach stems from its computational principle of learning a probabilistic cell‐to‐voxel mapping. Specifically, the model computes a mapping matrix, M, that defines the probability of each single cell being located in each spatial voxel. This matrix is optimized by minimizing a custom loss function that aligns scRNA‐seq and spatial transcriptomic data based on two criteria: (i) Gene Expression Similarity, assessed via cosine similarity to match expression patterns across genes and voxels, and (ii) Cell Density Similarity, constrained using *Kullback‐Leibler* (*KL*) divergence to incorporate prior knowledge on cell distribution per voxel. Through this optimization, the model spatially rearranges entire single‐cell transcriptomes to infer their most probable locations, thereby generating a high‐resolution, cell‐level map and enabling deconvolution of cellular mixtures as well as accurate imputation of spatial gene expression patterns.

The model underwent 2000 epochs with a learning rate of 0.01, operating in “CELLS” mode for cell‐to‐spot mapping, with spatial constraints enforced via *Density_prior = ‘rna_count_based*’. Optimization was performed using the Adam optimizer to minimize *KL* divergence between scRNA‐seq and spatial transcriptomic datasets. Cell annotations (Subclass_label) were projected onto spatial coordinates using *tg.project_cell_annotations*, with dominant cell types per spot determined via *tangram_ct_pred*. Integrated transcriptomes were generated using *tg.project_genes*, followed by rigorous quality assessment. Model performance was evaluated using *tg.plot_training_scores*, whil*e tg.plot_genes_sc* was used to compare spatial and integrated transcriptomes to validate low‐scoring gene expression patterns. Concordance between scRNA‐seq and integrated transcriptomes was further confirmed by calculating gene AUC scores (*tg.compare_spatial_geneexp*).

Following integration, the high‐resolution and spatially informed transcriptional atlas of GCs encompassing the developmental continuum from Type‐5b, Type‐6, and Type‐7/8 follicles were extracted. Upregulated genes were identified and cross‐referenced with the AnimalTFDB database (http://bioinfo.life.hust.edu.cn/AnimalTFDB/) to determine the upregulated TFs. Follicle staging criteria was established by the Pedersen and Peters [59]. Type‐5b follicles contain ≥5 GC layers surrounding a fully grown oocyte without a follicular antrum; Type‐6 follicles exhibit multilayered GCs separated by small, irregular antra; Type‐7 follicles possess a large antrum with a defined cumulus oophorus; and Type‐8 follicles display a large antrum with a well‐developed cumulus stalk.

### 4.5 Cell and Follicle Culture

Mouse primary GCs were aseptically isolated from large antral follicle 24 h post‐PMSG injection. Sheep primary GCs were aseptically isolated from small antral follicles (300–350 µm diameter). Human KGN GC line (Procell, China) was donated by Dr. A.L. (Huazhong Agricultural University, China). Mouse and sheep GCs were cultured in DMEM/F12 medium (Gibco, USA) supplemented with 10% fetal bovine serum (Gibco, USA), 10 mIU mL^−^^1^ FSH (NSHF, China) and 100 U mL^−^^1^ penicillin/streptomycin (Gibco, USA). KGN cells were cultured in DMEM/High medium (Gibco, USA) supplemented with 15% fetal bovine serum (Gibco, USA), 10 mIU mL^−^^1^ FSH (NSHF, China) and 100 U mL^−^^1^ penicillin/streptomycin (Gibco, USA). All the cells maintained at 37°C in a humidified atmosphere of 5% CO_2_.

Mouse small preantral follicles (150–180 µm diameter) and small antral follicles (250–300 µm diameter) were microdissected from mouse ovaries using 33‐gauge microneedles (KONSFI, China). Follicles were individually cultured in 96‐well plates (BKMAM, China) containing 50 µL of culture medium under mineral oil (Sigma‐Aldrich, USA) overlay. The culture medium consisted of α‐MEM (Gibco, USA) supplemented with 1% ITS‐G (Macklin, China), 5% FBS (Serana, Germany), 10 mIU mL^−^^1^ FSH (NSHF, China), 100 U mL^−^^1^ penicillin/streptomycin (Servicebio, China). The culture system was maintained at 37°C with 5% CO_2_. Following 120 h of culture of small preantral follicles, the small antra are formed. After 96 h of culture of small antral follicles, they develop to the pre‐ovulatory stage.

Sheep small antral follicles (300–350 µm diameter) were isolated from ovaries using ophthalmic scissors and 26‐gauge microneedles (KONSFI, China). Follicles were cultured in 96‐well plates containing 100 µL of culture medium under mineral oil overlay at 38.5°C with 5% CO_2_. The culture medium contained α‐MEM basal medium, 10% FBS, 1% ITS‐G, 50 µg mL^−^^1^ ascorbic acid (Sigma‐Aldrich, USA), 2 mM hypoxanthine (Sigma‐Aldrich, USA), 2 mM glutamine (Sigma‐Aldrich, USA), 10 mIU mL^−^^1^ FSH, 100 U mL^−^^1^ penicillin/streptomycin. Under these conditions, follicle diameter typically increased to approximately 500 µm after 96 h of culture.

The follicular radius (FR), follicular area (FA) and follicular antral area (FAA) were measured using ImageJ software (v1.52, NIH, USA) to calculate follicular volume (follicular volume = 4/3*π*FR^3^) and antral fraction (antral fraction = FAA/FA).

### 4.6 RNA Interference

*Fosl2*‐specific siRNA (Genepharma, China) was employed to silence *Fosl2* expression in cultured mouse GCs and KGN cells. Transfection was performed using jetPRIME transfection reagent (PolyPlus‐transfection, France) according to the manufacturer's protocol. Briefly, cells were transfected with 100 pmol of siRNA in culture medium for 48 h, followed by replacement with fresh culture medium for subsequent experiments.

Lentivirus‐mediated RNA interference was used to decrease the expression of *Fosl2* and *Creb* in cultured follicles or sheep GCs. The PLKO.1‐EGFP‐PURO plasmid (Genecreate, China) was used to construct shRNA interference vectors. Lentiviral particles were produced by co‐transfecting HEK293T cells (ATCC, USA) with 4.8 µg of interference vector, 2.4 µg of pMD2.G envelope plasmid (Addgene, USA), 3.6 µg of pSPAX2 packaging plasmid (Addgene, USA). Viral supernatants were collected 48 h post‐transfection, centrifuged at 4000 rpm for 10 min, and filtered through 0.45 µm polyvinylidene fluoride (PVDF) membranes (Sigma‐Aldrich, USA). Follicles and sheep GCs were then incubated with the prepared viral particles (titer: 1.25 × 10^7^ particles mL^−^^1^) in culture medium containing 10 µg mL^−^^1^ Polybrene (Sigma‐Aldrich, USA). Transfection durations were optimized as 48 h (mouse follicles and sheep GCs) and 96 h (sheep follicles), respectively. Transfection was confirmed by EGFP fluorescence visualization. Following transfection, samples were either immediately processed for phenotypic analysis or maintained in fresh culture medium for subsequent experiments. Non‐targeting siRNA were purchased from Sigma‐Aldrich. The siRNA target sequences were provided in Table S5.

### 4.7 HE Staining

Ovaries were fixed in 4% paraformaldehyde (Servicebio, China) for 24 h at 4°C, followed by standard paraffin embedding. Sections (5 µm thickness) were prepared using a rotary microtome (Leica, Germany). After deparaffinization and rehydration, the sections were stained with hematoxylin (Servicebio, China) for 5 min. Then the sections were stained with eosin (Servicebio, China) for 5 min and followed by dehydration with graded alcohol and clearing in xylene. Stained sections were examined under a microscope (Olympus, Japan) equipped with a digital camera. Morphometric analysis was performed using ImageJ software.

### 4.8 qRT‐PCR Assay

Total RNA from samples was extracted using TRIzol reagent (Takara, Japan). Reverse transcription was performed using the PrimeScript RT reagent kit (Takara, Japan). The qRT‐PCR was performed by CFX384 Real‐Time PCR System (Bio‐Rad, USA). Reaction system consists of 5 µL SYBR Green (Biosharp, China), 2 µL complementary DNA template, 250 nM of the forward and reverse primers for each, and ddH_2_O was supplemented to a total volume of 10 µL. The reaction protocol was conducted as described: an initial denaturation step at 95°C for 10 min, succeeded by 35 cycles comprising denaturation at 95°C for 10 s and annealing/ extension at 60°C for 30 s. A final step included a melting curve analysis ranging from 60°C to 95°C, with a 0.5°C increment every 5 s. Using the housekeeping gene *Actb* to normalize gene expression levels, and using comparative 2^−△△Ct^ method to determine relative RNA. The primer sequences used for PCR amplification are provided in Table S5.

### 4.9 Western Blot

Total protein was extracted using RIPA lysis buffer (ComWin Biotech, China) supplemented with protease and phosphatase inhibitor (ComWin Biotech, China). Protein concentrations were quantified using the BCA Protein Assay Kit (Servicebio, China) according to the manufacturer's protocol. Equal amounts of protein (typically 20–50 µg per lane) were separated by 10% SDS‐PAGE and subsequently transferred to PVDF membranes (0.45 µm pore size; Sigma‐Aldrich, USA) using standard wet transfer methods. Membranes were blocked with 5% non‐fat dry milk in Tris‐buffered saline containing 0.1% Tween‐20 (TBST) for 2 h at room temperature. Following blocking, membranes were incubated overnight at 4°C with the following primary antibodies diluted in blocking buffer: FOSL2 (1:1000, Sigma‐Aldrich, USA), Caspase‐3 (1:1000, CST, USA), cleaved‐Caspase‐3 (1:1000, CST, USA), BAX (1:1000, CST, USA), phospho‐PKA (1:1000, CST, USA), AKT (1:1000, CST, USA), phospho‐ AKT (1:1000, CST, USA), mTOR (1:1000, CST, USA), phospho‐mTOR (1:1000, CST, USA), ERK1/2 (1:1000, Servicebio, China), phospho‐ERK1/2 (1:1000, Abclonal, China), EPAC1 (1:1000, CST, USA), CREB (1:1000, CST, USA), phospho‐CREB (1:1000, CST, USA), α‐Tubulin (1:1000, Servicebio, China) and GAPDH (1:1000, Abclonal, China). After primary antibody incubation, membranes were washed three times (10 min each) with TBST and then incubated with horseradish peroxidase (HRP)‐conjugated secondary antibodies (goat anti‐rabbit IgG, 1:4000; goat anti‐mouse IgG, 1:4000, Biodragon‐immunotech, China) for 1 h at room temperature. Protein bands were visualized using an enhanced chemiluminescence (ECL) detection kit (Biosharp, China) according to the manufacturer's instructions, and images were captured using a ChemiDoc imaging system (Tanon‐5200, China). Band intensities were quantified using ImageJ software, with normalization to appropriate loading controls (α‐Tubulin or GAPDH).

### 4.10 Immunofluorescence

Ovarian tissues were sectioned at 5 µm thickness using a rotary microtome and mounted on poly‐L‐lysine‐coated slides (CITOTEST, China). After deparaffinization through xylene and graded ethanol series, antigen retrieval was performed in citrate buffer at 95–98 °C for 25 min. Sections were permeabilized with 0.5% Triton X‐100 in PBS for 20 min at room temperature, followed by blocking with 10% goat serum (Boster, China) in PBS for 60 min at room temperature. For immunostaining, sections were incubated with rabbit anti‐FOSL2 primary antibody (1:200 dilution, Sigma‐Aldrich, USA) at 4°C overnight, then with an HRP‐conjugated secondary antibody working solution (Recordbio Biological Technology, China) at 37°C for 50 min. Subsequently, the sections were treated with the fluorescent dye TYR‐520 (Recordbio Biological Technology, China) for 5 min, followed by a heat treatment to eliminate nonspecific dye binding. Dual‐staining of FOSL2 and FOXL2 was conducted using the Three‐color Fluorescence kit (Recordbio Biological Technology, China) based on Tyramide Signal Amplification technology. Briefly, following antigen blocking, the sections were incubated overnight at 4°C with rabbit anti‐FOSL2 primary antibody. After rinsing with PBS, the sections were incubated with an HRP‐conjugated secondary antibody working solution (Recordbio Biological Technology, China) at 37°C for 50 min. Subsequently, the sections were treated with the fluorescent dye TYR‐520 for 5 min, followed by eliminating nonspecific dye binding. The same protocol was applied for FOXL2 detection using the primary antibody against FOXL2 (1:100 dilution, Abcam, USA) and fluorescent dye TYR‐570 (Recordbio Biological Technology, China) for FOXL2 staining. Additionally, cell nuclei were stained with DAPI (Biosharp, China). Following another round of washing, the sections were imaged using an LSM800 confocal microscope system (Zeiss, Germany) and the resulting images were analyzed using Zen 2.3 lite software.

### 4.11 EdU Staining

GC proliferation was evaluated using the EdU assay kit (RiboBio, China). Following siRNA‐mediated gene silencing, mouse primary GCs and KGN cells grown on cell climbing slices (RiboBio, China) were labeled with 50 µM EdU at 37°C under 5% CO_2_ atmosphere for 2 and 4 h, respectively. Subsequently, GCs were fixed with 4% paraformaldehyde (Servicebio, China) for 30 min at room temperature. Permeabilization was achieved using 0.5% Triton X‐100 (Servicebio, China) in PBS for 10 min. The reaction was performed by incubating samples with EdU dye reaction solution for 30 min in the dark, followed by nuclear counterstaining with Hoechst 33342 (RiboBio, China) for 10 min. For follicle assessment, cultured follicles were incubated with 50 µM EdU for 24 h post‐plasmid transfection. Follicles were then embedded in optimal cutting temperature compound (OCT) (Sakura Finetek, Japan) and snap‐frozen in liquid nitrogen for 1 min. Cryosections (5 µm thickness) were obtained using a CM195 cryostat (Leica, Germany). The sections were processed for EdU detection using the same reaction conditions as for GCs, with 30‐min EdU staining and 10‐min Hoechst 33342 counterstaining. Fluorescent images were captured using a fluorescence microscope (Olympus, Japan). Quantitative analysis was performed using ImageJ software. Proportion of EdU‐positive nuclei = (EdU‐positive cells/total cells) × 100%.

### 4.12 Flow Cytometry Assay

The cell cycle distribution was assessed using a Cell Cycle Detection Kit (Keygentec, China). Following RNA interference, GCs were fixed in 70% ice‐cold ethanol at 4°C overnight. Fixed cells were then washed with PBS and stained with 500 µL Propidium Iodide (PI)/RNase A working solution for 60 min at room temperature in the dark. Cellular DNA content was measured by CytoFLEX LX System (Beckman, USA) with excitation at 488 nm and emission detection at 617 nm. Data analysis was performed using ModFit LT Software Version 5.0 (Verity Software House, USA) to determine the percentage of cells in G1, S, and G2/M phases.

Apoptotic cells were quantified using an Annexin‐V‐FITC/PI apoptosis detection kit (Beyotime, China). Briefly, GCs were resuspended in 195 µL Annexin‐V binding buffer and stained with 5 µL Annexin‐V‐FITC and 10 µL PI working solution for 15 min at room temperature in the dark. Flow cytometric analysis was immediately performed using the CytoFLEX LX system, with Annexin‐V‐FITC fluorescence detected in the FL1 channel (530/30 nm bandpass filter) and PI fluorescence in the FL3 channel (>670 nm longpass filter). Viable (Annexin‐V/PI), early apoptotic (Annexin‐V^+^/PI), late apoptotic (Annexin‐V^+^/PI^+^), and necrotic (Annexin‐V/PI^+^) cell populations were distinguished and quantified using FlowJo software (Leonard Herzenberg, USA).

### 4.13 EMSA Assay

The coding sequence of *Fosl2* was subcloned into the pcDNA3.1‐3XFlag expression vector (Addgene, USA) to generate Flag‐tagged FOSL2 protein. Following overexpression, Flag‐FOSL2 protein were immunoprecipitated using anti‐Flag monoclonal antibody (Beyotime, China) and subsequently eluted under acidic conditions (0.1 M glycine, pH 2.7), followed by immediate neutralization with 1 M Tris‐HCl (pH 8.5). JASPAR web‐based platform (https://jaspar.elixir.no/) was employed to predict the direct binding sites of FOSL2 protein within the promoter regions of the *Fshr, Cyp11a1* and *Cyp19a1*. Based on the predicted motifs, double‐stranded DNA probes and corresponding site‐directed mutant probes were designed, and subsequently synthesized by GENE CREATE company (Wuhan, China). EMSA was performed using the Chemiluminescent EMSA Kit (Beyotime, China). According to the manufacturer's instructions, biotin‐labeled double‐stranded DNA probes (Genecreate, China) were incubated with purified Flag‐FOSL2 protein in binding buffer for 30 min at room temperature to allow complex formation. Protein‐DNA interactions were resolved on a 4% non‐denaturing polyacrylamide gel at 100 V for 60 min. The resolved complexes were then electrophoretically transferred to Amersham Hybond‐N^+^ nylon membranes (Cytiva, USA) and detected using HRP‐conjugated streptavidin followed by autoradiography. The specific primer sequences used for probe generation were provided in Table S5.

### 4.14 Luciferase Reporter Assay

The promoter regions of *Fshr*, *Cyp11a1*, *Cyp19a1* and *Fosl2* were PCR‐amplified and directionally cloned into the pGL3‐Basic luciferase reporter vector (Promega, USA) using the ClonExpress Ultra One Step Cloning Kit (Vazyme, China). For over‐expression vector construction, the complete coding sequences of *Fosl2* and *Creb* were amplified and subcloned into pcDNA3.1‐3XFlag vectors (Addgene, USA). Mouse and sheep primary GCs were plated in 24‐well plates and cultured for 24 h prior to transfection. Cells were co‐transfected with 248 ng of over‐expression vector, 248 ng of pGL3‐based reporter construct, and 2.5 ng of pRL‐TK Renilla luciferase control vector (Promega, USA) using jetPRIME transfection reagent according to the manufacturer's protocol. After 24‐h incubation, cells were harvested in 100 µL passive lysis buffer and analyzed using the Dual‐Luciferase Reporter Assay System (Promega, USA). Firefly and Renilla luciferase activities were quantified sequentially using an EnSpire Multimode Plate Reader (PerkinElmer, USA). Relative promoter activity was calculated as the ratio of firefly to Renilla luciferase luminescence. All primer sequences used for cloning and mutagenesis are provided in Table S5.

### 4.15 Hormone Determination

Steroid hormone in serum or culture medium was quantitatively determined using a highly specific radioimmunoassay (RIA) system. All measurements were performed by the Beijing North Institute of Biological Technology (Beijing, China), a certified clinical testing laboratory, utilizing a commercially available RIA kit (Bioengineering Institute, China) with established sensitivity (E2: 10 pg mL^−^^1^, T: 0.1 ng mL^−^^1^). The assay employed competitive binding principles using ^125^I‐labeled hormone tracer and highly specific anti‐hormone antibodies, following the manufacturer's standardized protocol.

### 4.16 Statistics Analysis

Statistical analyses were using GraphPad Prism 10.0 (GraphPad). Data were expressed as the mean ± SD. Two‐tailed unpaired Student's t test and one‐way analysis of variance followed by Tukey's post hoc test were used to analyze the statistical significance between two groups and among multiple groups, respectively. Chi‐squared test was used in the comparison between the percentages. The statistical significance was set at *p*‐value <0.05.

## Author Contributions

C.H. conceived, designed, conducted the experiments, analyzed and interpreted the data; H.S., C.C., and Z.R. participated in experiment design and conduction, data analysis, and manuscript preparation; J.L., Z.W., X.W., Y.Z., W.K., B.T., and Y.L. assisted with sample collection and experiments conduction. Y.S. bred *Fshr‐Cre* mice. C.H. wrote the manuscript. C.H., H.S., C.C., Z.R., X.L., and W.R. improved the manuscript. C.H., X.L., and Y.S. funded this project. C.H., X.L., and W.R. supervised this project. All authors approved the final version.

## Funding

This research was supported by the Biological Breeding‐Major Projects in National Science and Technology (2023ZD04049) and the Fundamental Research Funds for the Central Universities (2662023DKPY001), the Hubei Agricultural Science and Technology Innovation Center (2021‐620‐000‐001‐030) and the National Natural Science Foundation of China (Grant Nos. 32330031, 32170860).

## Conflicts of Interest

The authors declare no conflicts of interest.

## Supporting information




**Supporting File 1**: advs75223‐sup‐0001‐SuppMat.docx.

**Supporting File 2**: advs75223‐sup‐0002‐TableS1.xlsx.

**Supporting File 3**: advs75223‐sup‐0003‐TableS2.xlsx.

**Supporting File 4**: advs75223‐sup‐0004‐TableS3.xlsx.

**Supporting File 5**: advs75223‐sup‐0005‐TableS4.xlsx.

## Data Availability

The RNA‐Seq data reported in this study have been deposited in the Gene Expression Omnibus database (GSE297300). All data are available from the corresponding author upon reasonable request.
