# Supplementary material for: Fosl2 Regulates FSH‐Dependent Follicle Maturation Through Feedback Amplification of FSH/FSHR Signaling
Source: Adv Sci (Weinh). 2026 Apr 9:e75223. Online ahead of print. doi: 10.1002/advs.75223 (PMC13334666; doi:10.1002/advs.75223)
Supplement: Supplementary file 1 — Supporting File 1: advs75223‐sup‐0001‐SuppMat.docx. [file ADVS-9999-e75223-s002.docx]

Supporting Information

***Fosl2* regulates FSH-dependent** **follicle development through feedback amplification of FSH/FSHR signaling**

Hongru Shi^1^^Ϯ^, Chaoli Chen^2Ϯ^, Zaohong Ran^1Ϯ^, Jianning Liao^1^, Zian Wu^1^, Xiaodong Wang^1^, Yongheng Zhao^1^, Wenkai Ke^1^, Bowen Tan^1^, Yun Liu^1^, Youqiang Su^3^, Wei Ren^2*^, Xiang Li^1*^, Changjiu He^1*^

^Ϯ^ H. S., C. C., and Z. R. contributed equally to this work.

*Corresponding author. Email: chungjoe@mail.hzau.edu.cn

**
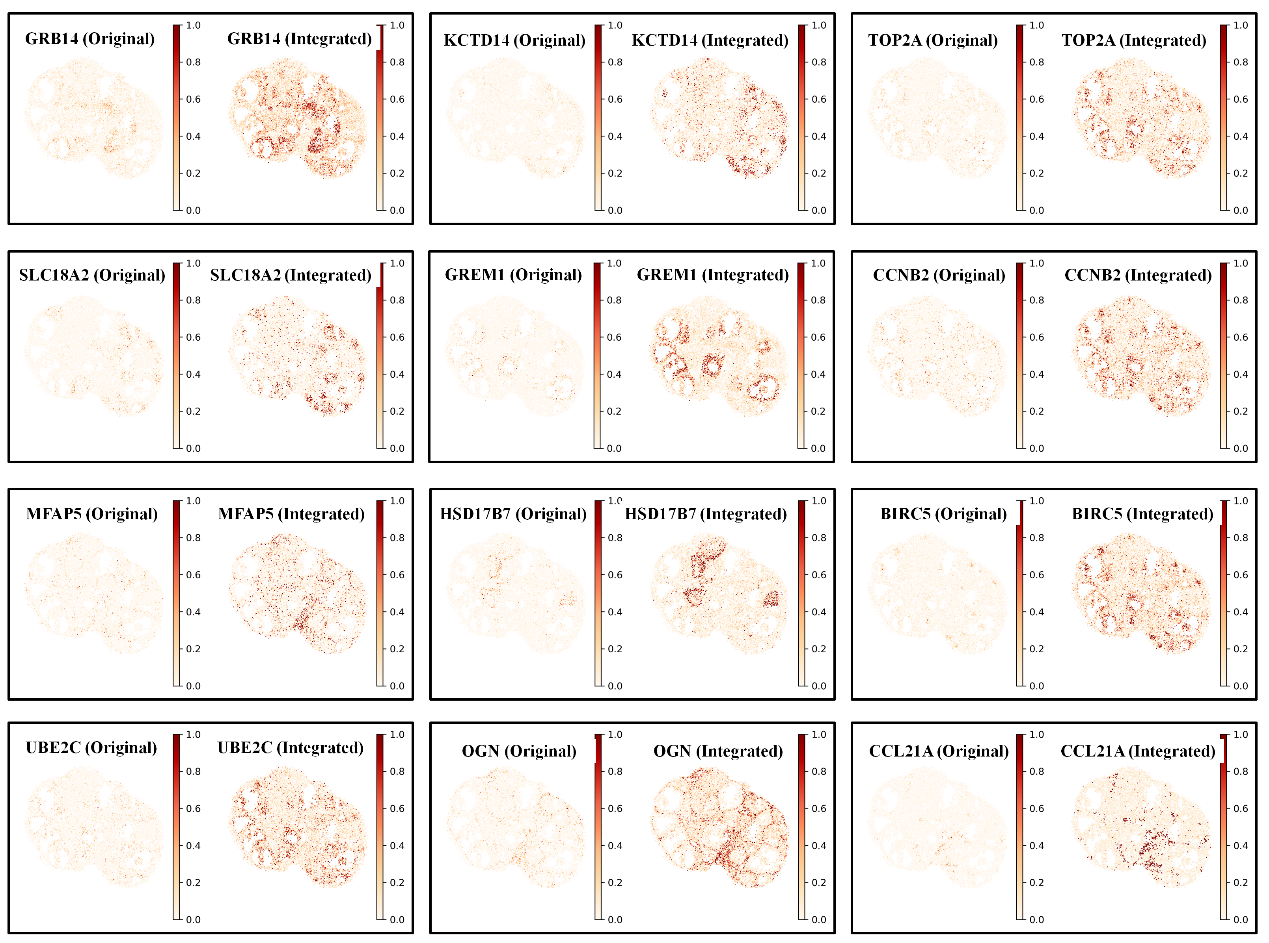
**

**Figure S1. Expression analysis of randomly selected genes showed that the integrated spatial dataset exhibited higher resolution and more distinct spatial localization compared to the original spatial transcriptomics dataset (related to Figure 1).**

**
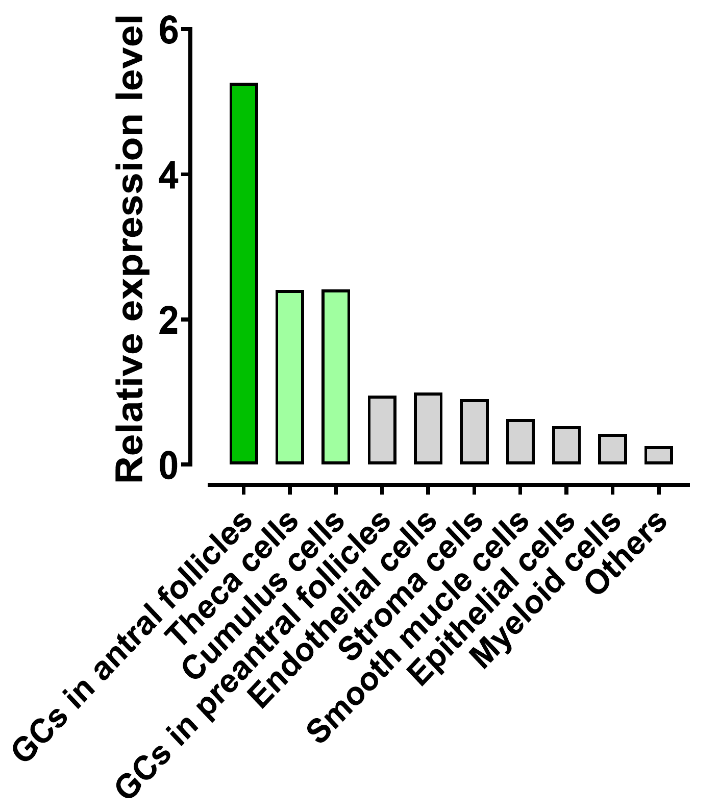
**

**Figure S2. Quantitative analysis of *Fosl2* expression across ovarian cell types using single-cell transcriptomics (related to Figure 1).** Ovaries were collected from 21-day-old Kunming mice 48 hours post-injection with PMSG.

**
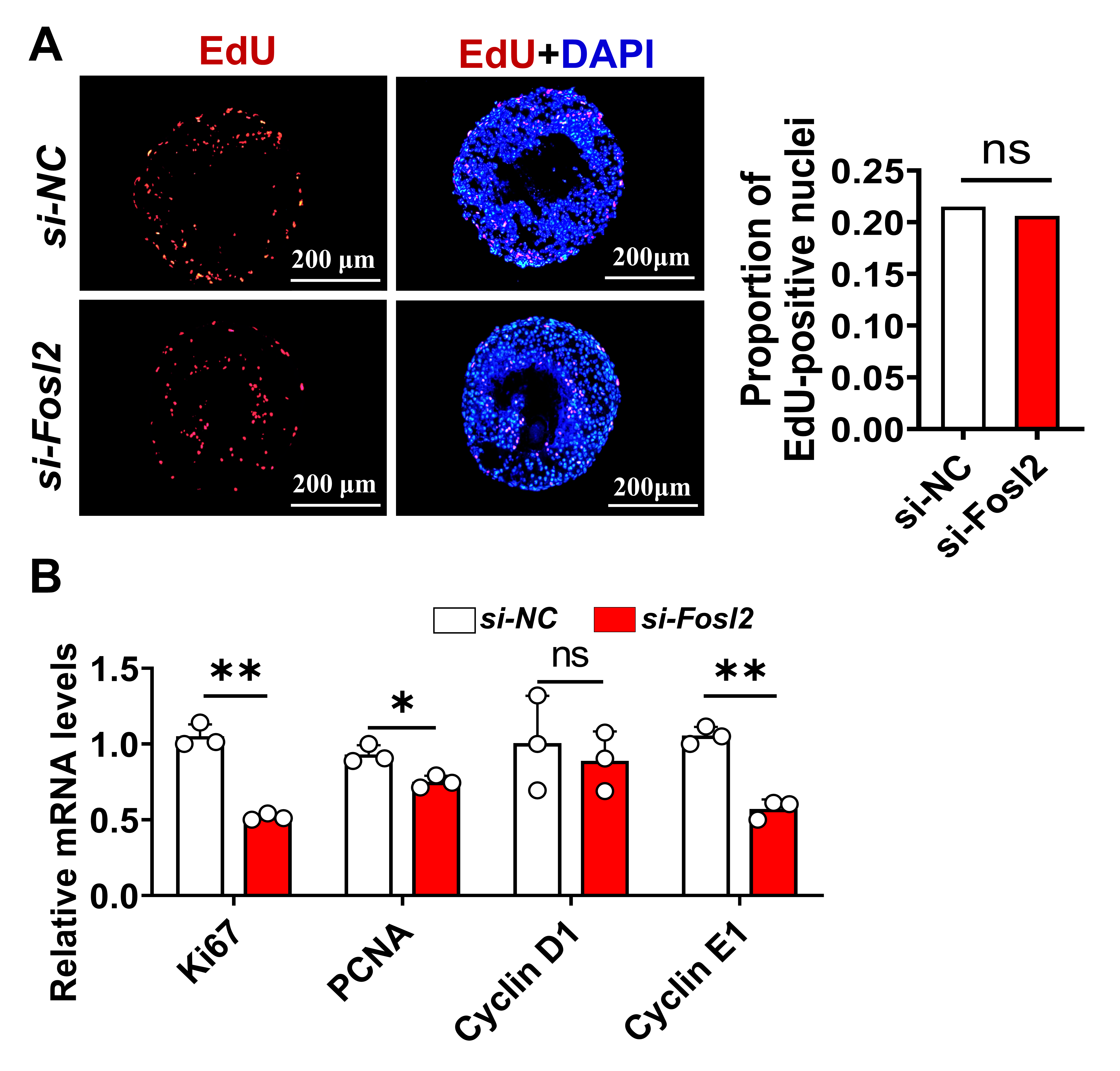
**

**Figure S3. Knockdown of *Fosl2* plays a different role at distinct developmental stages** **(related to Figure 3).** (A) Follicular cell proliferation analysis using the EdU incorporation assay. Left: representative images of EdU staining; right: quantification of EdU-positive nuclei, n = 4 follicles. (B) Expression analysis of proliferation-related genes using qRT-PCR, n = 3 follicles. Statistical significance was determined using two-tailed unpaired Student’s t-test or chi-square test, values were mean ± SD. Signiﬁcant differences were denoted by *P<0.05, **P<0.01. Shown is a representative result from three independent experiments with similar outcomes.

**
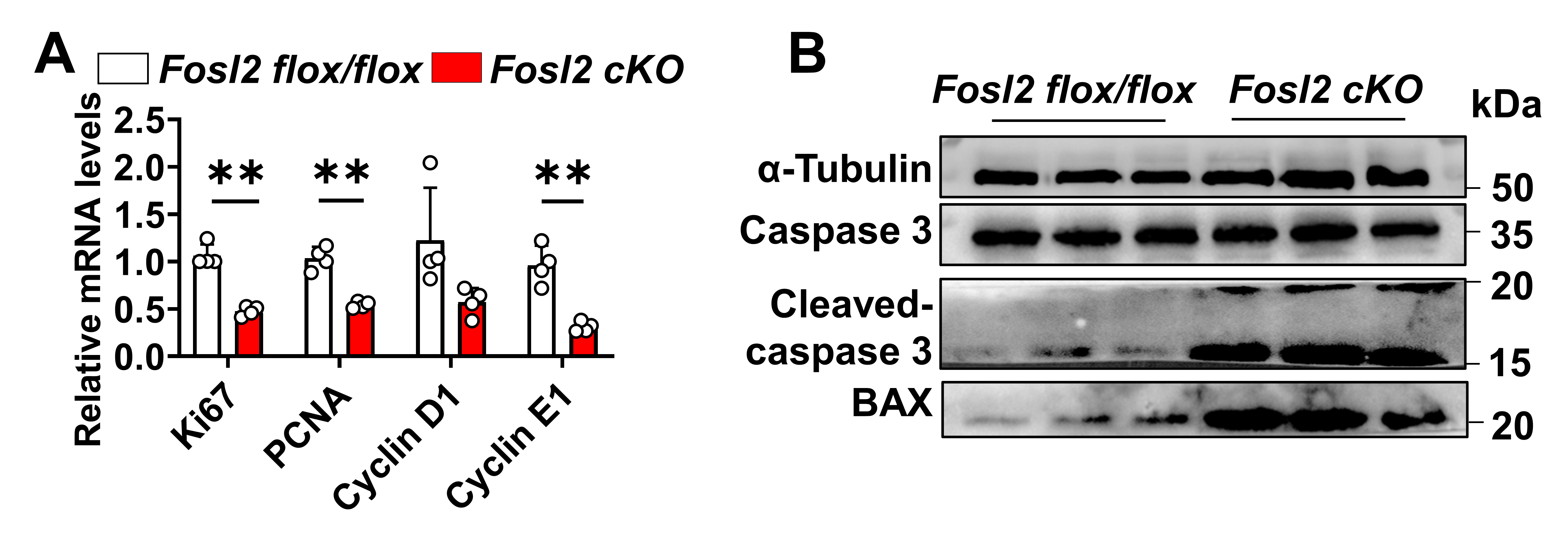
**

**Figure S4.** **GC-specific *Fosl2* knockout** **impaired GC proliferation and induces apoptosis (related to Figure 4).** (A) Expression analysis of proliferation-associated genes using qRT-PCR, n = 4 GC samples. (B) Changes in pro-apoptotic protein contents following *Fosl2* knockout, n = 3 GC samples. GCs were isolated from ovaries 48 hours post-PMSG injection. Original blots were provided in Figure S9. Statistical signiﬁcance were determined using one-way ANOVA followed by Tukey’s post hoc test, values were mean ± SD. Signiﬁcant differences were denoted by **P<0.01. Shown is a representative result from three independent experiments with similar outcomes.


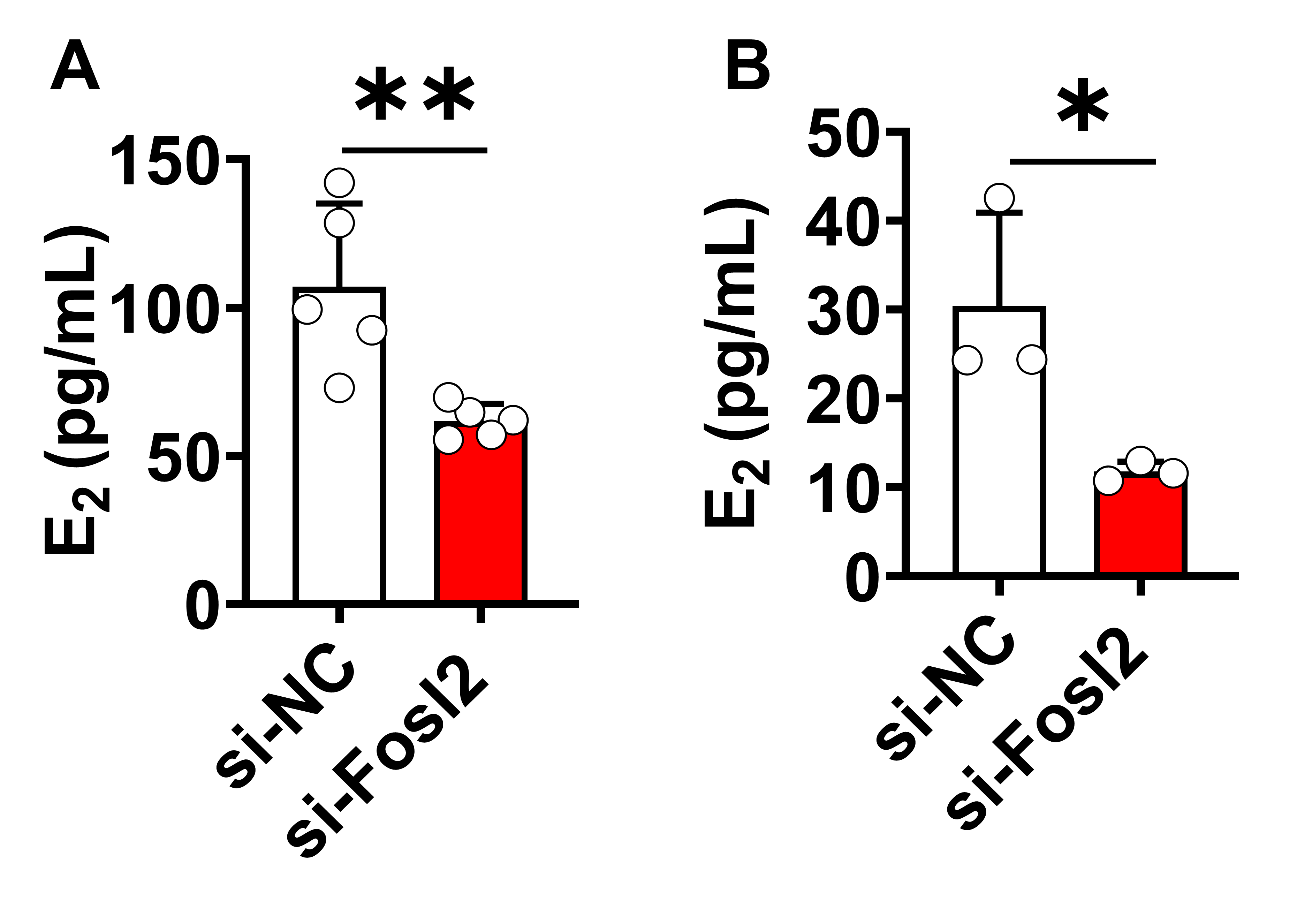


**Figure S5. Knockdown of *Fosl2* impaired estrogen biosynthesis (related to Figure 5).** (A) Effect of *Fosl2* knockdown in mouse primary GCs on estradiol (E_2_) secretion into the culture medium, n = 5. (B) Effect of *Fosl2* knockdown in FSH-dependent follicles on E_2_ secretion into the culture medium, n = 3. Statistical signiﬁcance were determined using one-way ANOVA followed by Tukey’s post hoc test, values were mean ± SD. Signiﬁcant differences were denoted by *P<0.05, **P<0.01. Shown is a representative result from three independent experiments with similar outcomes.

**Figure S6. Knockdown of *Fosl2* impaired estrogen biosynthesis-related genes in human KGN GC line (related to Figure 5).** qRT-PCR analysis of *Fshr*, *Cyp11a1*, and *Cyp19a1* expression in human KGN GC line; n = 3 GC samples. Statistical signiﬁcance were determined using one-way ANOVA followed by Tukey’s post hoc test, values were mean ± SD. Signiﬁcant differences were denoted by *P<0.05, **P<0.01. Shown is a representative result from three independent experiments with similar outcomes.

**
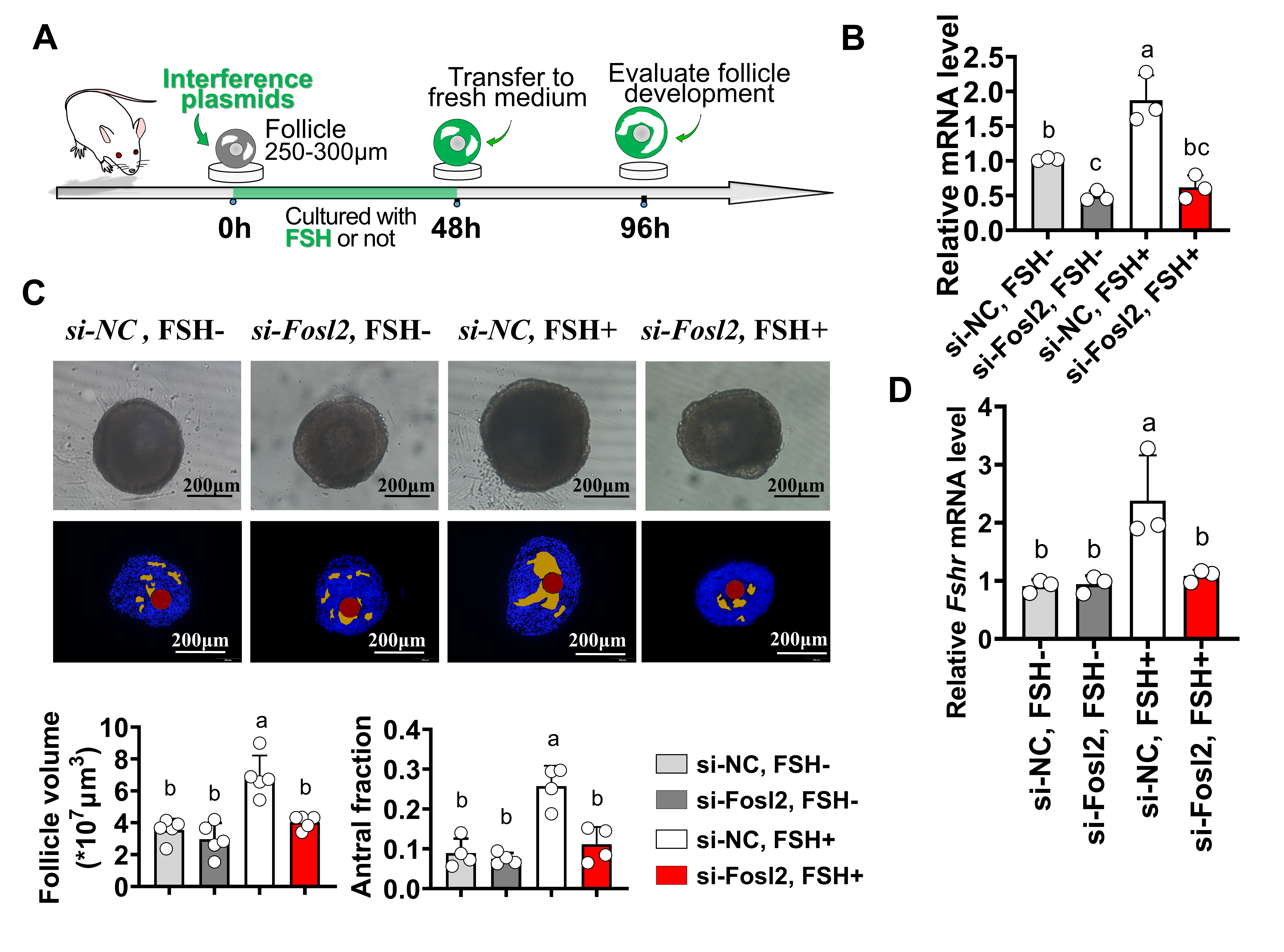
**

**Figure S7. *Fosl2* is required for the upregulation—but not the basal expression—of *Fshr*.** (A) Schematic representation illustrating the experimental design. (B) qRT-PCR analysis of *Fosl2* expression following disruption of *Fosl2* in the presence or absence of FSH. n = 3 follicular samples. (C) Follicle volume changes and alterations in follicular antral fraction following *Fosl2* knockdown in the presence or absence of FSH ; n = 5 follicles (follicle volume), n = 4 follicles(antral fraction). The yellow area indicates the follicular antrum. Red area represents the oocyte. (D) qRT-PCR analysis of *Fshr* expression following disruption of *Fosl2* in the presence or absence of FSH. n = 3 follicular samples. Data are presented as mean ± SD and are representative of two independent experiments showing similar results. Statistical significance was assessed by one-way ANOVA followed by Tukey’s post hoc test. Different letters (a-c) denote significant differences between groups.

**
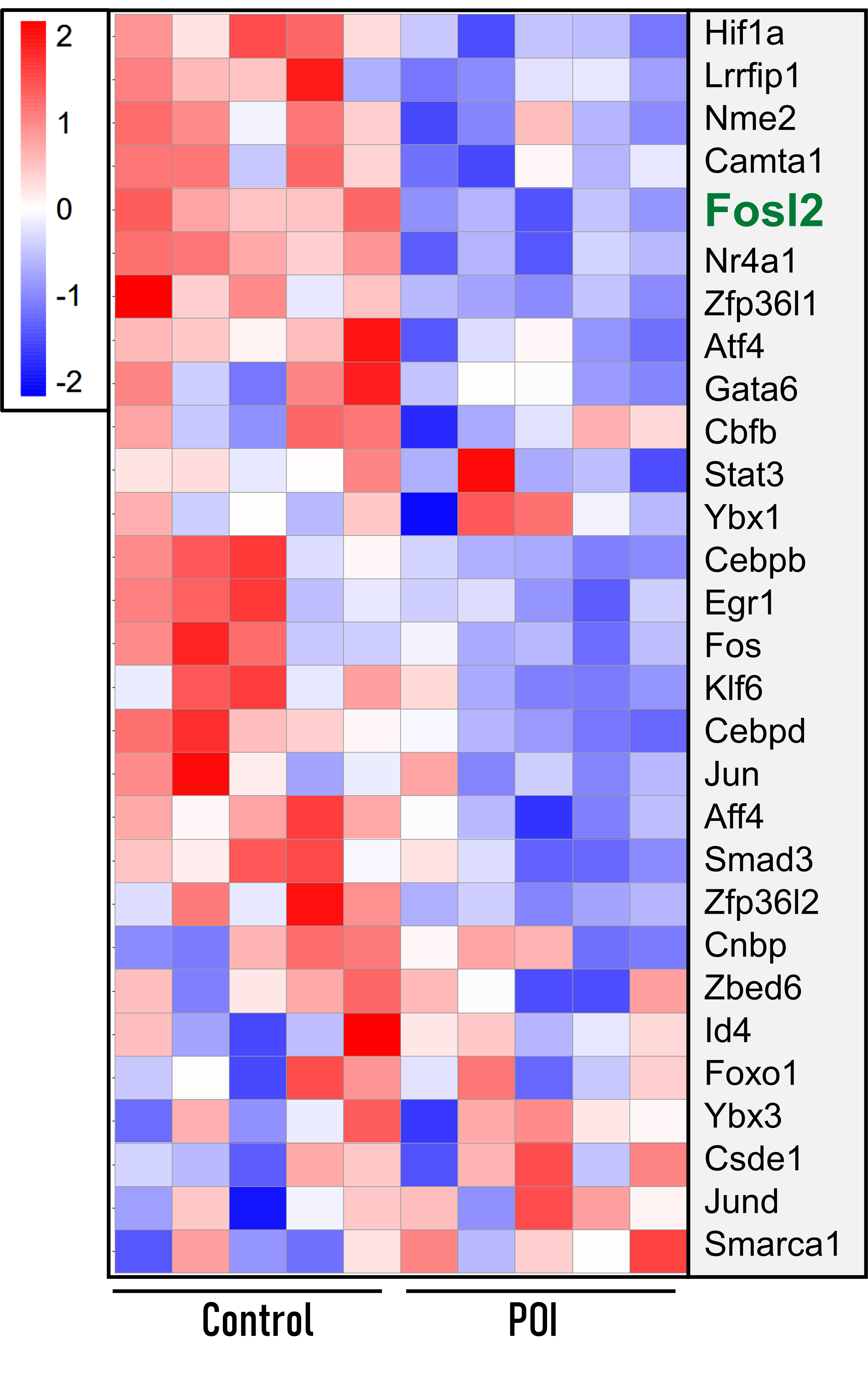
**

**Figure S8.** **Heatmap of downregulated TFs in GCs of patients with premature ovarian insufficiency (POI).**

**
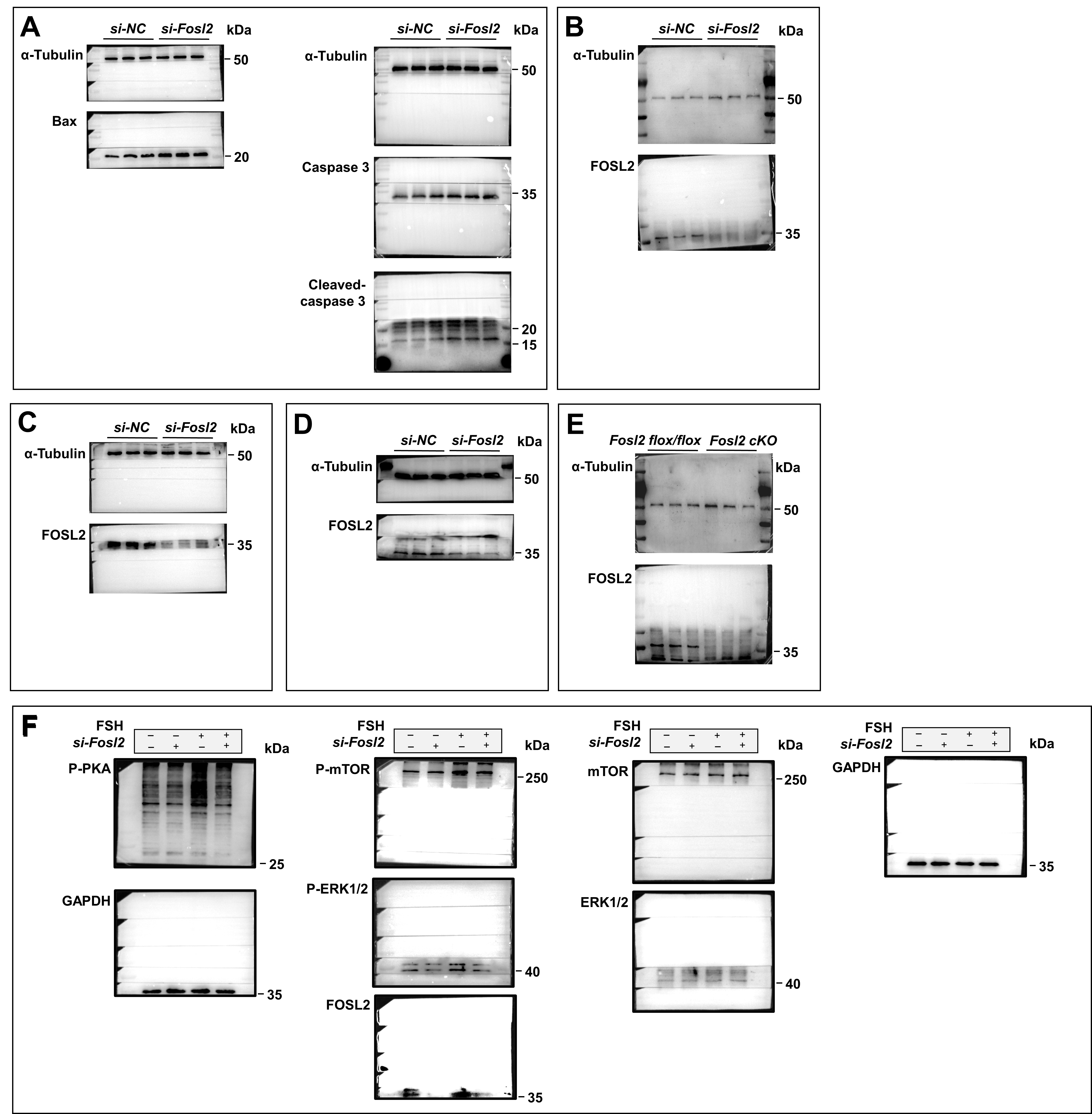
**

**Figure S9. Full western blots.** (A) Western blot analysis of pro-apoptotic proteins BAX and cleaved Caspase-3 in mouse GCs after *Fosl2* knockdown; n = 3 GC samples (related to Figure 2F). (B) Western blot analysis of FOSL2 protein levels in mouse FSH-independent follicles following *Fosl2* knockdown (related to Figure 3B). (C) Western blot analysis of FOSL2 protein levels in mouse FSH-dependent follicles following *Fosl2* knockdown (related to Figure 3F). (D) Western blot analysis of FOSL2 protein levels in sheep FSH-dependent follicles following *Fosl2* knockdown (related to Figure 3K). (E) Western blot analysis of FOSL2 protein levels in *cKO* mice (related to Figure 4C). (F) FSH-downstream signaling cascades in *si-Fosl2* GCs following FSH supplement (related to Figure 5G).

**
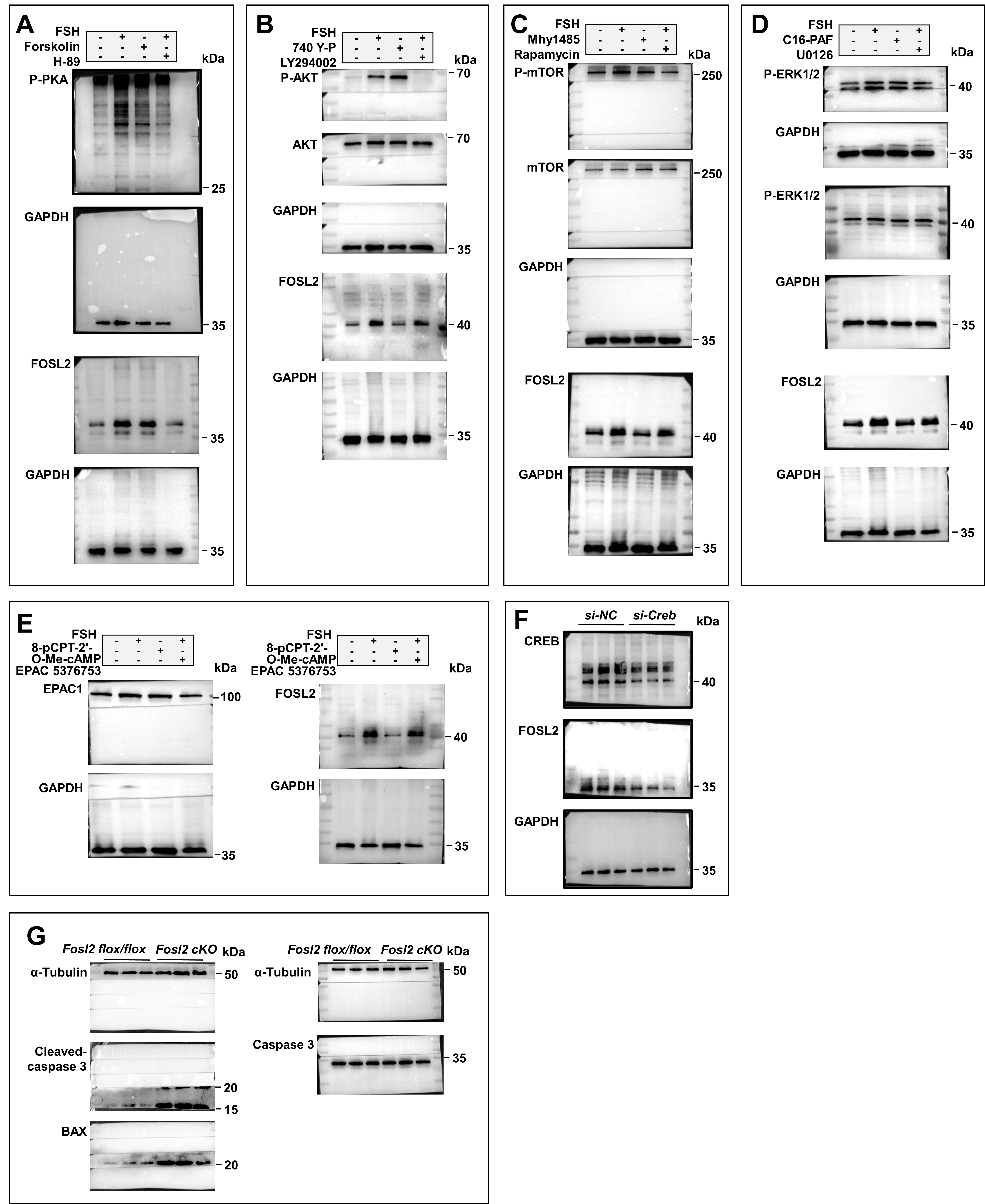
**

**Figure S10. Full western blots.** (A) Western blot analysis of FOSL2 protein levels in GCs following activation or inhibition of the cAMP-PKA cascade (related to Figure 6C). (B) Western blot analysis of FOSL2 protein levels in GCs following activation or inhibition of the PI3K-AKT cascade (related to Figure 6C). (C) Western blot analysis of FOSL2 protein levels in GCs following activation or inhibition of the mTOR cascade (related to Figure 6C). (D) Western blot analysis of FOSL2 protein levels in GCs following activation or inhibition of the ERK cascade (related to Figure 6C). (E) Western blot analysis of FOSL2 protein levels in GCs following activation or inhibition of the EPAC cascade (related to Figure 6C). (F)Western blot analysis of FOSL2 and CREB protein levels in GCs following *Creb* knockdown (related to Figure 6E). (G) Western blot analysis of pro-apoptotic protein levels in *cKO* mice (related to Figure S4B).

**Table S5. The primers used for qPCR and Luciferase reporter**

| Gene | Primer sequence (5'-3') |
| --- | --- |
| *β-Actin-mouse* | Forward: CCAGCCTTCCTTCTTGGGTAT |
|  | Reverse: AGGTCTTTACGGATGTCAACG |
| *Fosl2-mouse* | Forward: GGTAGATATGCCTGGCTCGG |
|  | Reverse: TCATCTCTCCTTCTGCGGCC |
| *Ki67-mouse* | Forward: ATCATTGACCGCTCCTTTAGGT |
|  | Reverse: GCTCGCCTTGATGGTTCCT |
| *Cyclin D1-mouse* | Forward: GCTGGTAGTATGAGGTGCTTGG |
|  | Reverse: CTTTGCAGGACAGATCCCG |
| *Cyclin E1-mouse* | Forward: AGACAAAGCCCAAGCAAAGAA |
|  | Reverse: TGGAGGCAATGGCAGGTT |
| *PCNA-mouse* | Forward: ACCTGCAGAGCATGGACTCG |
|  | Reverse: GCAGCGGTATGTGTCGAAGC |
| *Fshr-mouse* | Forward: TGCTCTAACAGGGTCTTCCTC |
|  | Reverse: TCTCAGTTCAATGGCGTTCCG |
| *Cyp11a1-mouse* | Forward: AGGTCCTTCAATGAGATCCCTT |
|  | Reverse: TCCCTGTAAATGGGGCCATAC |
| *Cyp19a1-mouse* | Forward: ATGGGCCTCCTTCTCCTGAT |
|  | Reverse: CAGGCACTTCCAATCCCCAT |
| *β-Actin-sheep* | Forward: CCTGCGGCATTCACGAAACTAC |
|  | Reverse: ACAGCACCCTGTTGGCGTAGAG |
| *Fosl2-sheep* | Forward: CGGGAACTTTGACACCTCGT |
|  | Reverse: TGATGGCGTTGATGGTAGGG |
| *Cyclin B1-sheep* | Forward: TGGCTACTITCCACTTGAGGAT |
|  | Reverse: GGTGACTTGGGCTTACACACA |
| *Cyclin D1-sheep* | Forward: GCTGGTCCTGGTGAACAAAC |
|  | Reverse: CACAGAGGGCAACGAAGGTC |
| *Cyclin E1-sheep* | Forward: AGATGCGCACAACATCCAGA |
|  | Reverse: CAAAGTGAAGAGGCTGCCCA |
| *PCNA-sheep* | Forward: TCTCATGTCTCCTTGGTGCA |
|  | Reverse: GCCAAGGTGTCCGCATTATC |
| *Fshr-sheep* | Forward: ATGCGGTCGAACTGAGGTTT |
|  | Reverse: GCAGGTTGTTGGCCTTTTCA |
| *Cyp11a1-sheep* | Forward: GTTTCGCTTTGCCTTTGAGTC |
|  | Reverse: ACAGTTCTGGAGGGAGGTTGA |
| *Cyp19a1-sheep* | Forward: CCCCAGGTTGAAGAGGCAAT |
|  | Reverse: CTGGTACCGCATGCTCTCAT |
| *Fosl2-human* | Forward: TGACGTTCGTCAGACCCTTG |
|  | Reverse: CTTCTCCTCCTCCAGCTCCT |
| *Fshr-**human* | Forward: GAGGCCTTCCAGAACCTTCC |
|  | Reverse: ACTTTCAAAGCTCAGCCCCA |
| *Cyp11a1-human* | Forward: GCAGTGTCTCGGGACTTCG |
|  | Reverse: GGCAAAGCGGAACAGGTCA |
| *Cyp19a1-human* | Forward: ATGAAAGCTCTGTCAGGCCC |
|  | Reverse: TCAACACGTCCACATAGCCC |
| *Fosl2-Cyp11a1* | Forward: AGAGGAGGGATGACTCTTGT |
|  | Reverse: ACAAGAGTCATCCCTCCTCT |
| *Mut-FOSL2-Cyp11a1* | Forward: AGAGGAGGGAGCACGCTTGT |
|  | Reverse: ACAAGCGTGCTCCCTCCTCT |
| *Fosl2-Fshr* | Forward: CCTTTAGTGGGTCACGTGAC |
|  | Reverse: GTCACGTGACCCACTAAAGG |
| *Mut-Fosl2-Fshr* | Forward: CCTTTAGGCGGCCACGTGAC |
|  | Reverse: GTCACGTGGCCGCCTAAAGG |
| *Fosl2-Cyp19a1* | Forward: TGTCTGAGACTCACCCAGTCTG |
|  | Reverse: CAGACTGGGTGAGTCTCAGACA |
| *Mut-Fosl2-Cyp19a1* | Forward: TGTCGCAGACGCACCCAGTCTG |
|  | Reverse: CAGACTGGGTGCGTCTGCGACA |
| *INFU-PGL3* | Forward: GCTTGGCATTCCGGTACTGT |
|  | Reverse: GCTAGCACGCGTAAGAGCTC |
| *INFU-Fshr* | Forward: gagctcttacgcgtgctagcACAAGAACAAACTTCAGATTAGCTGC |
|  | Reverse: acagtaccggaatgccaagcAGGCTCCTCCACACACTGTCC |
| *INFU-Cyp11a1* | Forward: gagctcttacgcgtgctagcTAGCATGCATACAGCCCTGAGT |
|  | Reverse: acagtaccggaatgccaagcCGATGCACCGTTCACTCTCC |
| *INFU-Cyp19a1* | Forward: gagctcttacgcgtgctagcCCCCAGGACCTATGGGAAAC |
|  | Reverse: acagtaccggaatgccaagcACCCTGTCCTTTGGGCCTT |
| *INFU-sheep Fshr* | Forward: gagctcttacgcgtgctagcCTTGGAAGAAAGGTTATGACCAACC |
|  | Reverse: acagtaccggaatgccaagcGTGACCCGCCCAGGGCCG |
| *INFU-sheep Cyp11a1* | Forward: gagctcttacgcgtgctagcAGTCTGAACATCAAAATGATATAATTAAAGG |
|  | Reverse: acagtaccggaatgccaagcATGACCCCAGATTCTTGCACA |
| *INFU-sheep Cyp19a1* | Forward: gagctcttacgcgtgctagcTGATATGTACAACAAAGTGTTAATGTCCA |
|  | Reverse: acagtaccggaatgccaagcAAAGTCCTGTGGAGATCAGAAGAGTG |
| *INFU-Fosl2* | Forward: gagctcttacgcgtgctagcCTGCTCCCCTTCTCCCCC |
|  | Reverse: acagtaccggaatgccaagcGTGGATCCACTCACGTCAACCT |
| *si-Fosl**2-mouse* | Sense: GGCACUUCAAACCUUGUCUTT |
|  | Anti-sense: AGACAAGGUUUGAAGUGCCTT |
| *si-Fosl2-sheep* | Sense: GAACCUCGTCTUCACCTACTI |
|  | Anti-sense: GUAGGUGAAGACGAGGUUCTT |
| *si-Fosl2-human* | Sense: GGCCCAGUGUGCAAGAUUATT |
|  | Anti-sense: UAAUCUUGCACACUGGGCCTT |
| *si-Creb-mouse* | Sense: CAGCAGCUCAUGCAACAUCAUTT |
|  | Anti-sense: AUGAUGUUGCAUGAGCUGCUGTT |
